# Single-cell transcriptomic analysis reveals gut microbiota-immunotherapy synergy through modulating tumor microenvironment

**DOI:** 10.1038/s41392-025-02226-7

**Published:** 2025-05-02

**Authors:** Minyuan Cao, Yun Deng, Qing Hao, Huayun Yan, Quan-Lin Wang, Chunyan Dong, Jing Wu, Yajiao He, Li-Bin Huang, Xuyang Xia, Yongchao Gao, Hai-Ning Chen, Wei-Han Zhang, Yan-Jing Zhang, Xiaozhen Zhuo, Lunzhi Dai, Hongbo Hu, Yong Peng, Feng Zhang, Zhaoqian Liu, Weihua Huang, Huiyuan Zhang, Li Yang, Yang Shu, Wei Zhang, Yan Zhang, Heng Xu

**Affiliations:** 1 Department of Laboratory Medicine/Research Centre of Clinical Laboratory Medicine, State Key Laboratory of Biotherapy, West China Hospital, Sichuan University, Chengdu, China; 2 State Key Laboratory of Biotherapy and Cancer Center, West China Hospital, Sichuan University, Chengdu, China; 3 Department of Clinical Pharmacology, Xiangya Hospital, Central Laboratory of Hunan Cancer Hospital, Central South University, Changsha, China; 4 Division of Gastrointestinal Surgery, Department of General Surgery, West China Hospital, Sichuan University, Chengdu, China; 5 Colorectal Cancer Center, Department of General Surgery, West China Hospital, Sichuan University, Chengdu, China; 6 Gastric Cancer Center, Department of General Surgery, West China Hospital, Sichuan University, Chengdu, China; 7 Core Facilities, West China Hospital, Sichuan University, Chengdu, China; 8 Department of Cardiology, The First Affiliated Hospital, Xi’an Jiaotong University, Xi’an, China; 9 Center for Precision Medicine, The Quzhou Affiliated Hospital of Wenzhou Medical University, Quzhou People’s Hospital, Quzhou, China; 10 The First Affiliated Hospital of Guangdong Pharmaceutical University, Guangzhou, China; 11 Lung Cancer Center/Lung Cancer Institute, Department of Medical Oncology, West China Hospital, Sichuan University, Chengdu, China; 12 Institute of General Surgery, West China Hospital, Sichuan University, Chengdu, China; 13 Tianfu Jincheng Laboratory, Chengdu, China

**Keywords:** Cancer microenvironment, Tumour immunology

## Abstract

The gut microbiota crucially regulates the efficacy of immune checkpoint inhibitor (ICI) based immunotherapy, but the underlying mechanisms remain unclear at the single-cell resolution. Using single-cell RNA sequencing and subsequent validations, we investigate gut microbiota-ICI synergy by profiling the tumor microenvironment (TME) and elucidating critical cellular interactions in mouse models. Our findings reveal that intact gut microbiota combined with ICIs may synergistically increase the proportions of *CD8*^+^, *CD4*^+^, and γδ T cells, reduce glycolysis metabolism, and reverse exhausted *CD8*^+^ T cells into memory/effector *CD8*^+^ T cells, enhancing antitumor response. This synergistic effect also induces macrophage reprogramming from M2 protumor *Spp1*^+^ tumor-associated macrophages (TAMs) to *Cd74*^+^ TAMs, which act as antigen-presenting cells (APCs). These macrophage subtypes show a negative correlation within tumors, particularly during fecal microbiota transplantation. Depleting *Spp1*^+^ TAMs in *Spp1* conditional knockout mice boosts ICI efficacy and T cell infiltration, regardless of gut microbiota status, suggesting a potential upstream role of the gut microbiota and highlighting the crucial negative impact of *Spp1*^+^ TAMs during macrophage reprogramming on immunotherapy outcomes. Mechanistically, we propose a γδ T cell-APC-*CD8*^+^ T cell axis, where gut microbiota and ICIs enhance Cd40lg expression on γδ T cells, activating Cd40 overexpressing APCs (e.g., *Cd74*^+^ TAMs) through CD40-CD40L-related NF-κB signaling and boosting *CD8*^+^ T cell responses via CD86-CD28 interactions. These findings highlight the potential importance of γδ T cells and *SPP1*-related macrophage reprogramming in activating *CD8*^+^ T cells, as well as the synergistic effect of gut microbiota and ICIs in immunotherapy through modulating the TME.

## Introduction

Immune checkpoint inhibitors (lCls) targeting PD-1/PD-L1 or CTLA-4 have fundamentally transformed cancer treatment paradigms, achieving durable clinical responses across diverse malignancies, including melanoma, non-small cell lung cancer, and mismatch repair-deficient colorectal cancer. However, only 15–40% of patients derive long-term benefits from ICI monotherapy, with substantial interpatient heterogeneity in treatment outcomes that remain poorly understood.^1,2^ This variability highlights the urgent need to decipher the complex biological networks governing therapeutic response and resistance. Mechanistically, ICls function by reinvigorating exhausted *CD8*^+^ T cells through the blockade of inhibitory checkpoints, thereby restoring antitumor immunity.^3^ However, emerging evidence suggests that T cell reinvigoration alone is insufficient to explain the full spectrum of clinical responses, pointing to critical contributions from other immune compartments and systemic factors. Efforts to understand the underlying mechanisms and identify potential biomarkers for ICI efficacy and resistance have revealed a multitude of factors operating across multiple biological scales. These include clinical variables (e.g., tumor mutational burden), host factors (e.g., germline genetics), tumor-intrinsic mechanisms (e.g., oncogenic signaling), and the tumor microenvironment (TME).^2,4,5^ Recent advances highlight the TME as a critical orchestrator of lCl resistance, where myeloid-derived suppressor cells, regulatory T cells (Tregs), and alternatively activated macrophages collectively establish an immune-evasive niche through overlapping immunosuppressive mechanisms.^6^ Among these, tumor-associated macrophages (TAMs) polarized toward an M2-like phenotype have emerged as key mediators of T cell dysfunction through multiple pathways, including PD-L1 expression, arginase-mediated nutrient deprivation, and secretion of anti-inflammatory cytokines like lL-10.^7^ Beyond direct immune suppression, M2-like TAMs promote angiogenesis through VEGF secretion and facilitating extracellular matrix remodeling via matrix metalloproteinases, processes that collectively reinforce therapeutic resistance by creating physical barriers to drug penetration and immune cell trafficking.^8^

A paradigm-shifting discovery in immuno-oncology has been the recognition of gut microbiota as a systemic modulator of ICI efficacy, bridging intestinal ecology with systemic antitumor immunity. Clinical and preclinical studies demonstrate that antibiotic-mediated depletion of gut bacteria diminishes ICI responses in both patients and mouse models.^9–14^ In contrast, fecal microbiota transplantation (FMT), either with specific bacteria species (e.g., *Akkermansia muciniphila, Bifidobacterium spp*.) or pooled microbiota from ICI responders, can enhance response rates and the efficacy of ICI-based therapies, suggesting a potential synergistic role between ICIs and the gut microbiota in immunotherapy.^10,11,15^ Dysbiosis of gut microbiota and their metabolites (e.g., short-chain fatty acids, inosine) can directly influence systemic immunity by altering TME composition.^16^ For instance, FMT combined with PD-1 blockade promotes intratumoral *CD8*^*+*^ T cell infiltration and tumor necrosis.^15^ Moreover, specific gut bacteria species can enhance effector T cell function in the peripheral blood and the TME, potentially augmenting the antitumor efficacy of ICIs.^17^ These bacteria can also activate *CD4*^+^ T cells and reduce Treg cell proportions in peripheral blood, which may contribute to the long-term clinical benefits of ICI.^18^ Mechanistically, the gut microbiota may influence the ICI response through several pathways, including stimulation of DC and interferon (IFN) γ^+^ CD8^+^ T cells by metabolic byproducts (e.g., inosine) of specific bacteria species,^19^ monocytes reprogramming in the TME,^20^ and ligand-receptor communications (e.g., PD-L2-RGMb) between DCs and T cells in lymph nodes.^21^

With the development of single-cell RNA-sequencing (scRNA-seq) technology and its rapid application in tumor biology,^22^ the TME can now be systematically deconvoluted at cellular resolution, facilitating the understanding of its altered proportion of different cell types, tumor heterogeneity, molecular pathways, and cell-cell communications.^23^ In lCl-treated patients, scRNA-seq has identified clonally expanded *CD8*^+^ T cell subsets with stem-like properties predictive of response, as well as immunosuppressive TAM populations enriched in non-responders.^24–28^ However, patient heterogeneity and confounding variables in clinical cohorts present challenges for making comparisons across different treatment groups to reveal the potential mechanisms by which ICI and gut microbiota regulate the TME. In murine systems, several studies have focused on specific cell types and identified their regulatory roles through scRNA-seq and subsequent experimental validation. For instance, microbiota-induced STING-type I IFN-dependent monocyte reprogramming of the TME has been demonstrated,^20^ and *SPP1*^+^ tumor-associated macrophages (TAMs) have been identified as a determinant of immunotherapy efficacy involved in the tumor immune barrier.^25^ However, scRNA-seq-based investigation into the synergistic effects of gut microbiota and ICI treatment remains limited, particularly regarding the role of cell-cell communications.

In this study, we employed scRNA-seq coupled with flow cytometry, and multiplex immunofluorescence to dissect microbiota-ICI synergistic role in tumor models. Using a 2 × 2 factorial design (anti-PD-1 ± antibiotics), we demonstrate that intact gut microbiota potentiates lCl efficacy through three interlinked mechanisms: First, by expanding effector-memory *CD8*^+^ T cells while restraining terminal exhaustion; Second, microbial signals drive macrophage lineage commitment away from *SPP1*^+^ protumoral macrophages toward *CD74*^+^ antigen-presenting states. Third, microbiota-dependent activation of a γδ T cell-APC-*CD8*^+^ T cell axis establishes a feedforward loop of immune activation via CD40L-CD40/NF-κB signaling. Crucially, conditional knockout of *Spp1* in macrophages abolished microbiota dependence of ICl response, identifying *SPP1*^+^ TAMs as pivotal targets for microbiota-mediated TME remodeling. Our findings provide a high-resolution roadmap of microbiota-immune crosstalk, offering rational strategies to overcome ICl resistance through microbiome modulation.

## Results

### Single-cell TME profile modulated by PD-1 Inhibitor and gut microbiota

**Fig. 1:**
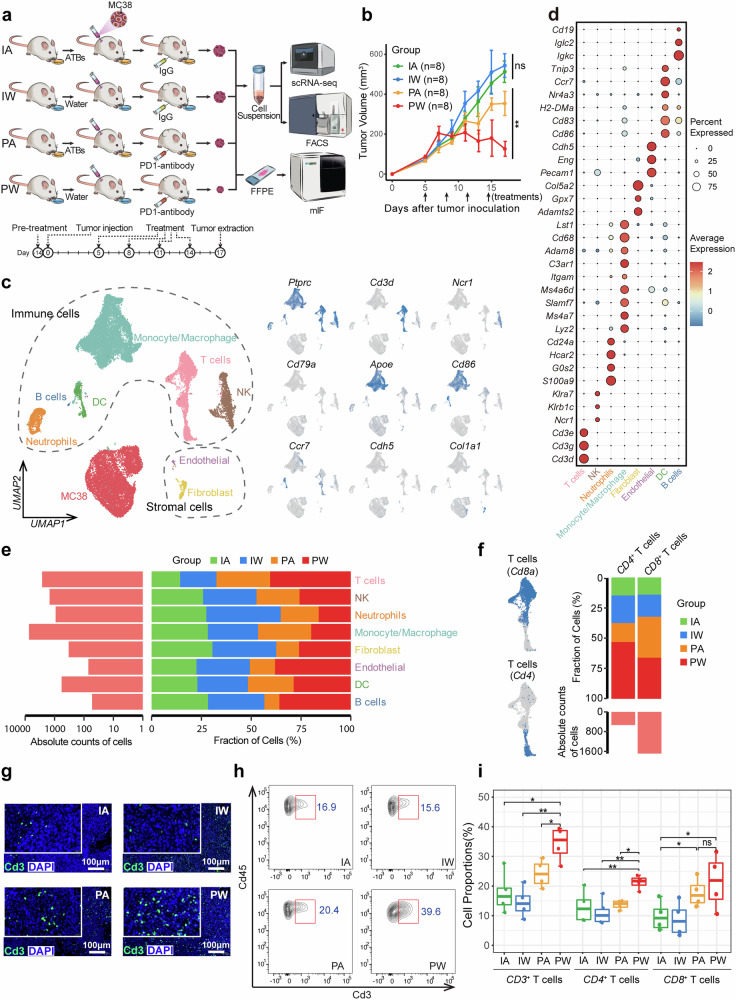
Single-cell profile of TME shaped by the gut microbiota and ICI treatment. **a** Schematic representation of the experimental design for scRNA-seq and experimental validations across four groups with different treatment strategies. **b** Line graph depicts the volumetric progression of subcutaneous tumors across four treatment groups. Each group consists of 8 mice (two batches combined). Data are represented as mean ± SEM. **c** The UMAP projection on the left illustrates cellular subpopulations, with dashed lines demarcating immune and stromal cells. The UMAP on the right highlights the expression of canonical genes for the identified clusters. **d** Bubble plot indicating the expression patterns of canonical marker genes for cellular clusters. **e** Bar graph showing the proportional representation of cell types across four groups. **f** The UMAP on the left highlights the expression of Cd4 and Cd8a in the T cell cluster. The bar graph on the right presents the proportional distribution of *CD4*^+^ and *CD8*^+^ T cells across four groups. **g** Multiplex immunofluorescence (mIF) imagery revealing the spatial distribution and abundance of Cd3 labeled T cells across four groups. **h** Flow cytometric analysis charting the relative abundance of T cells across four groups. **I** Box-and-whisker plots comparing the flow cytometry determined proportions of *CD3*^+^ T cells, *CD4*^+^ T cells, and *CD8*^+^ T cells across four groups. Each group consists of 4 mice

To investigate the interplay between gut microbiota and anti-PD-1 therapy, We implanted MC38 cells subcutaneously to establish tumor models and examined the synergistic effect of gut microbiota and immunotherapy using broad-spectrum antibiotics (ATBs) (see “Materials and methods”) and a PD-1 inhibitor. Mice maintained under specific pathogen-free (SPF) conditions were divided into four groups: IA (IgG + ATBs), IW (IgG + water), PA (PD-1 inhibitor + ATBs), and PW (PD-1 inhibitor + water). We performed scRNA-seq, flow cytometry, and multiplex immunofluorescence (mIF) to assess the impact of different treatments on the tumor microenvironment (TME) (Fig. 1a). ATBs treatment depleted the gut microbiota (Supplementary Fig. 1a), and tumor volume was significantly controlled by PD-1 inhibitor treatment in the PW group but only slightly reduced in the PA group compared to IA and IW groups (Fig. 1b and Supplementary Fig. 1b), supporting the crucial role of gut microbiota in ICI-based immunotherapy, consistent with previous reports.^11,15,17,19,29^ As validation, CT26 cells also exhibited sensitivity to PD-1 inhibitor treatment when initiated with a smaller tumor volume,^30^ while the efficacy was significantly diminished upon depletion of the gut microbiota (Supplementary Fig. 1c). Furthermore, PD-1 inhibitor treatment appeared to alter the proportion of certain gut microbiota species (e.g., *Akkermansiaceae*) but did not significantly impact the α- and β-diversity (Supplementary Fig. 1d–f).

Using scRNA-seq with pooled tumor samples post-treatment from each group and our established analysis pipeline,^31–35^ a total of 27,289 cells passed quality control and were divided into nine major cell types through unsupervised clustering. These cell types were annotated as immune cells, stromal cells, and tumor cells based on canonical makers and inferCNV-based copy number analysis (Fig. 1c, d and Supplementary Fig. 1g). The proportion of different TME cell components varied across the four groups, with increased T cells observed in mice treated with the PD-1 inhibitor, regardless of gut microbiota depletion (Fig. 1e and Supplementary Fig. 1h). Subtype analysis revealed that *CD8*^+^ T cells followed the same trend as total T cells, whereas *CD4*^+^ T cells were only significantly increased in the PW group, which responded well to the PD-1 inhibitor (Fig. 1f). These findings were validated in an independent batch of mice through mIF, flow cytometry, and immunohistochemistry, as well as in CT26-derived tumor (Fig. 1g–i and Supplementary Fig. 2).

### Impact of PD-1 inhibitor and gut microbiota on T cell subtypes

**Fig. 2:**
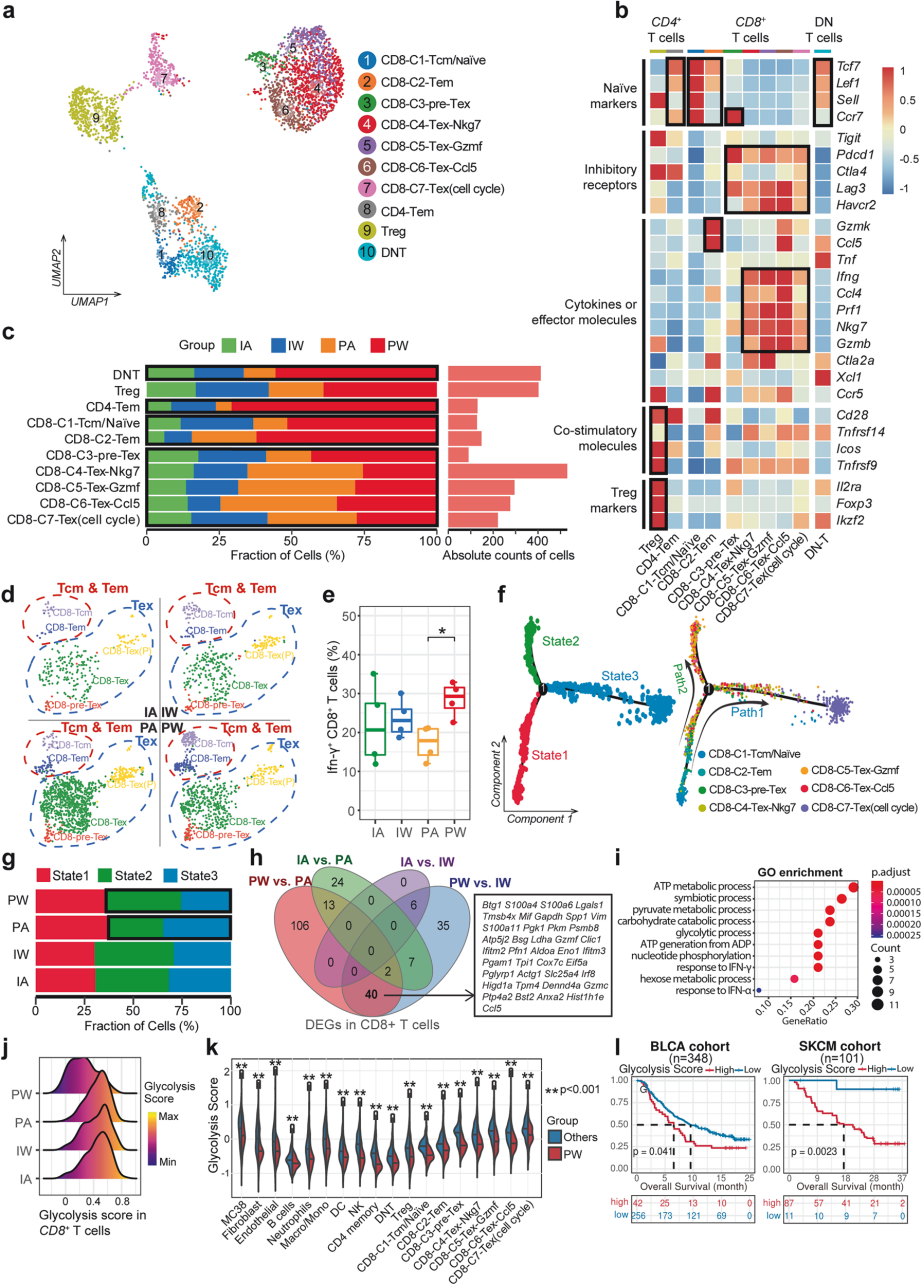
Characterization of subclusters of tumor-infiltrating T cells among different treatment groups. **a** UMAP visualization delineates the clustering and subtypes of T cells across four groups. **b** Heatmap illustrates the expression patterns of signature genes associated with T cell subtypes and functions. **c** Bar chart shows the proportional distribution of T cell subtypes across four groups. **d** UMAP plot reveals the distribution and proportion of *CD8*^+^ Tcm and *CD8*^+^ Tex cells across four groups. **e** Box-and-whisker plot represents the flow cytometry-based proportion of IFN-γ^+^ T cells across four treatment groups. Each group consists of tumor samples from 4 mice. **f** Trajectory plot reveals the evolutionary paths and distinct states of *CD8*^+^ T cells. **g** Bar chart indicates the proportions of *CD8*^+^ T cells in each state across four groups. **h** Venn diagram displays the intersecting differentially expressed genes (DEGs) of *CD8*^+^ T cells from four groups, with a focus box highlighting 40 DEGs influenced by both gut microbiota and aPD1 treatment in the PW group. **i** Bubble chart shows significant enriched functional pathways of the 40 DEGs. **j** Ridge plot depicts the glycolytic levels of *CD8*^+^ T cells across four groups. **k** Violin plot compares the glycolysis levels between the PW group cells and those from the others. **l** Kaplan–Meier survival curves demonstrate the relationship between cellular glycolytic levels and treatment outcomes in two immunotherapy cohorts

Given the crucial and diverse roles of tumor-infiltrating T cell subtypes in immunotherapy, we classified T cells into 10 subsets based on canonical makers, including seven *CD8*^+^, two *CD4*^+^, and one double-negative (*CD4*^−^*CD8*^−^) T cell subtypes (Fig. 2a and Supplementary Fig. 3a). Among *CD8*^+^ T cells, a central memory/naïve cell subgroup (CD8-C1-Tcm/naïve), an effector memory cell subgroup (CD8-C2-Tem), and five exhausted T cell subgroups were identified, characterized by the expression of T cell co-inhibitory receptors (e.g., *Pdcd1*, *Ctla4*, *Lag3*, and *Havcr2*) and cytotoxicity-related genes (e.g., Ifn-γ and Nkg7) (Fig. 2a, b and Supplementary Fig. 3a). Interestingly, the *CD8*^+^-C1-Tcm/naïve, *CD8*^+^-C2-Tem, *CD8*^+^-C3-pre-Tex (a precursor exhausted T cell), and *CD4*^+^ memory T cells were prominently enriched in responders (i.e., the PW group), while the proportions of exhausted T cell subtypes were higher in tumors treated with the PD-1 inhibitor (i.e., both the PA and PW groups) (Fig. 2c, d and Supplementary Fig. 3b). These findings highlight a potential correlation between cytotoxic and pre-exhausted *CD8*^+^ cells and the antitumor effects of immunotherapy. Additionally, the PW group showed an increased proportion of Ifn-γ^+^ cells compared to the PA group (Fig. 2e and Supplementary Fig. 3c). Trajectory inference analysis of all *CD8*^+^ T cells revealed two main evolutionary branches across three states (Fig. 2f), with the PA group showing more T cells in the terminally exhausted state (state 3), enriched with *CD8*^+^-C7-Tex (Fig. 2f, g). Furthermore, through CIBERSORT-based deconvolution with public bulk transcriptome data from three immunotherapy cohorts, including bladder cancer (BLCA), melanoma, and gastric cancer (STAD)^36,37^, the proportions of *CD8*^+^ effector/memory T cells and *CD4*^+^ memory T cells correlated with favorable prognosis and were enriched in responders (Supplementary Fig. 3d–f). These findings suggest dynamic *CD8*^+^ T cell exhaustion during ICI treatment, with gut microbiota potentially influencing the balance between effector/memory and exhausted T cells.

On the other hand, we compared transcriptional profiles of *CD8*^+^ T cells across the four treatment groups and found 40 differentially expressed genes (DEGs) influenced by the combination of PD-1 inhibitor and gut microbiota (Fig. 2h). These DEGs were enriched in multiple pathways, including ATP synthesis and glycolysis (Fig. 2i and Supplementary Fig. 4a). The PW group exhibited significantly lower glycolysis level than the other three groups (Fig. 2j), consistent with higher expression of *Gabpa* and *Bhlhe40* (Supplementary Fig. 4b), which support mitochondrial function.^38,39^ This lower glycolysis score was observed across all TME cell components in the PW group, aligning with public melanoma cohort data (Fig. 2k, and Supplementary Fig. 4c, d).^40^ Moreover, patients with higher deconvoluted glycolysis scores had poorer prognosis in two immunotherapy cohorts with follow-up information (i.e., BLCA and melanoma) (Fig. 2l). These findings suggest that the combination of PD-1 inhibitor and gut microbiota may synergistically influence tumor glycolysis-related energy metabolism and subsequently impact the efficacy of ICI-based immunotherapy.

### Profile of tumor-infiltrating double-negative T cells (DNT)

**Fig. 3:**
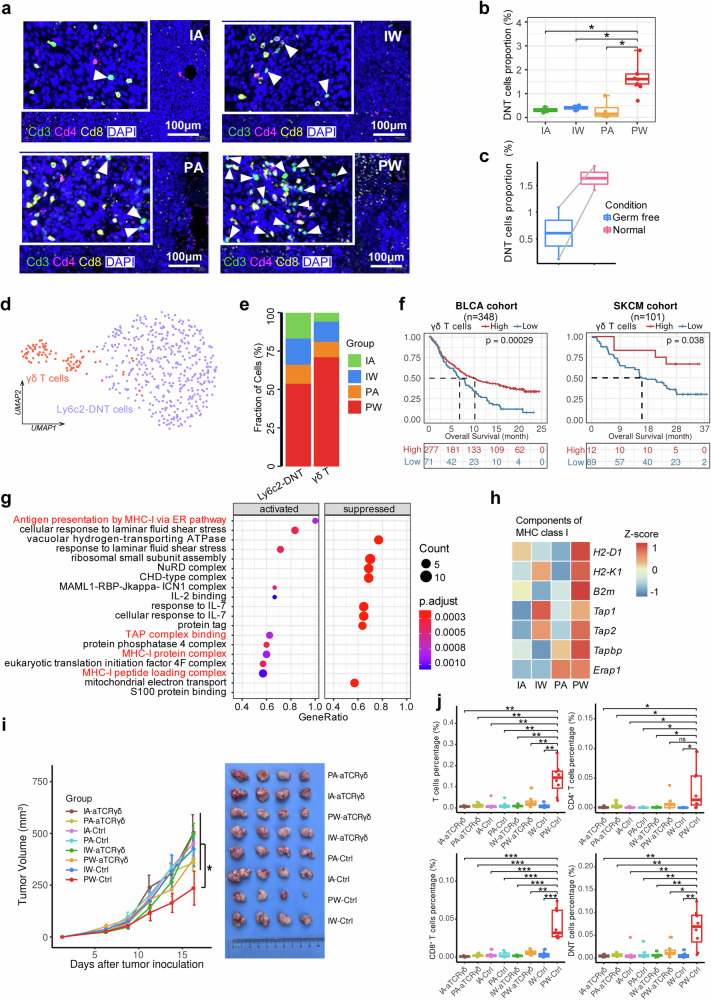
Synergistic effects of gut microbiota and ICI treatment on double-negative T (DNT) cells. **a** Representative mIF images illustrate the abundance of DNT cells (*CD3*^+^/*CD4*^−^/*CD8*^−^ T cells) within subcutaneous tumors across four groups. **b** Box plots depict the distribution and median counts of tumor-infiltrating DNT cells in subcutaneous tumors across four groups. Each group comprises four tumor samples, with data collected from two sections per tumor. **c** Comparison of tumor-infiltrating DNT cells in normobiotic mice with those germ-free counterparts based on GSE181745 dataset. **d** UMAP visualization delineates the clustering and subtypes of DNT cells. **e** Bar chart depicts the proportional distribution of DNT cell subtypes across four groups. **f** Kaplan–Meier survival curves associating tumor-infiltrating γδ T cell abundance with immunotherapy response. **g** Bubble chart depicts the functional enrichment analysis of gene sets in γδ T cells derived from subcutaneous tumors in the PW group compared to the others. **h** Heatmap of MHC-I related gene expression in tumor-infiltrating γδ T cells across four groups. **i** Line graph depicts the volumetric progression of subcutaneous tumors across eight groups. Each group consists of 4 mice. Data are represented as mean ± SEM. **j** Box-and-whisker plot represents the proportion of T cell subtypes quantified by multiplex immunofluorescence within the subcutaneous tumors of each mouse group, four tumor samples in each group, with data derived from two sections per sample

Besides *CD4*^+^ and *CD8*^+^ T cells, the proportion of DNTs was also higher in the PW group compared to the other groups (Fig. 2c), which can be validated through mIF (Fig. 3a, b, and Supplementary Fig. 5a). Using public mouse model data,^20^ we observed higher numbers of tumor-infiltrating DNTs in normobiotic mice compared to germ-free counterparts, supporting the role of gut microbiota in increasing DNT populations (Fig. 3c). We further divided DNTs into two subsets based on specific markers: Ly6c2-DNT (*Ly6c2*, *Ccl5*, and *Gramd3*) and γδ T cell (*Trdc*, *Tcrg-C1*, *Trdv4* and *Cd163l1*) (Fig. 3d and Supplementary Fig. 5b, c). The PW group was enriched in γδ T cells, suggesting a potential synergistic effect of immunotherapy and gut microbiota on γδ T cell induction (Fig. 3e). Functionally, γδ T cells bridge the innate and adaptive immune systems and are primarily located at the interface between tissues and the external environment (e.g., intestines). As such, they may continuously interact with commensal and pathogenic bacteria, potentially influencing ICB-based immunotherapy outcomes.^41^ Consistently, patients with higher CIBERSORT-based proportions of tumor-infiltrating γδ T cells correlated with better ICI response and prognosis in three immunotherapy cohorts (Fig. 3f and Supplementary Fig. 5d), aligning with recent evidence highlighting the crucial role of γδ T cells in modulating immunotherapy outcomes.^42,43^ Besides the increased γδ T cell proportions, DEGs in the PW group were enriched in multiple GSEA-based pathways, including the significantly activated MHC-I antigen presentation and antigen processing complex binding pathway (Fig. 3g and Supplementary Fig. 5e). Expression of multiple genes involved in MHC-I components and the MHC-I scores in γδ T cells were specifically higher in the PW group than the other groups (Fig. 3h and Supplementary Fig. 5f). Experimentally, depleting γδ T cells using a TCRγδ antibody substantially compromised the efficacy of the PD-1 inhibitor, along with the significantly decreased infiltrating T cells (Fig. 3i, j and Supplementary Fig. 6). These findings suggest that the γδ T cells may be functionally regulated by gut microbiota and involved in cross-presentation of tumor antigens, which could play a crucial role in immunotherapy.^44,45^

### Impact of gut microbiota and ICI treatment on myeloid cells

**Fig. 4:**
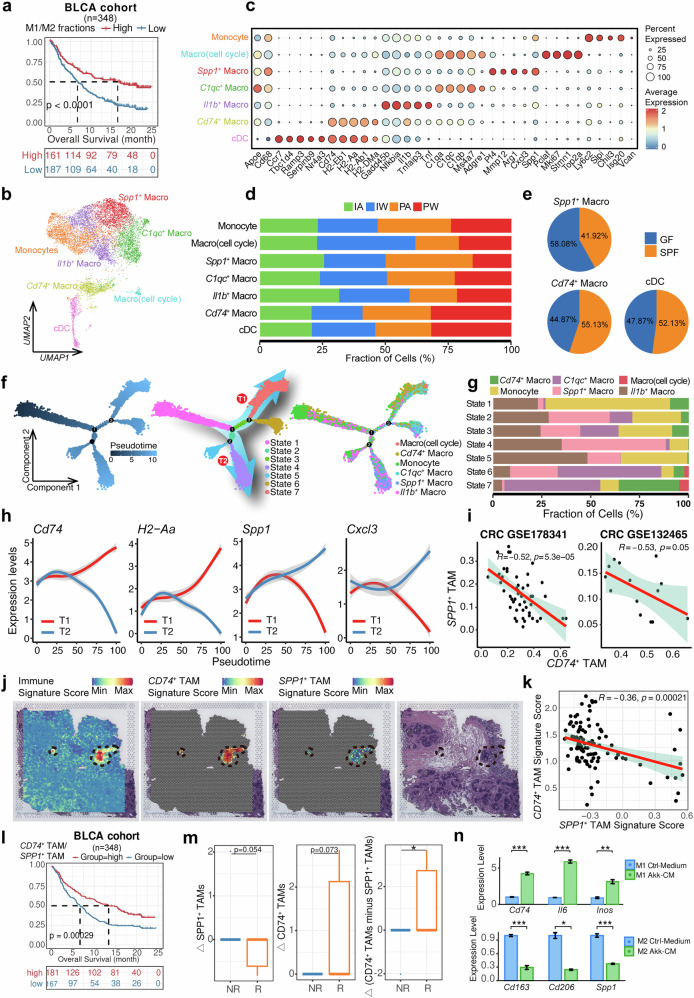
Characterization of myeloid cells and macrophage reprogramming upon ICI treatment. **a** Kaplan–Meier survival curve demonstrates the correlation of M1/M2 ratio with immunotherapy treatment outcomes in the bladder cancer (BLCA) cohort. **b** UMAP analysis delineates the clustering and subtypes of myeloid cells. **c** Bubble plot reveals the expression profiles of marker genes across myeloid cell subtypes. **d** Bar chart shows the proportional distribution of myeloid subtypes across four groups. **e** Pie chart shows the proportions of *Spp1*^+^ tumor-associated macrophages (TAMs), *Cd74*^+^ TAMs and cDC in normobiotic mice with their germ-free counterparts based on GSE181745 dataset. **f** Trajectory plot reveals the evolutionary paths and distinct states of macrophages/monocytes. **g** Bar graph shows the proportions of monocyte/macrophage subtypes across 7 evolutionary states. **h** Line graph reveals loess-regression-smoothened expression of the antigen-presenting (i.e., *Cd74* and *H2-Aa*) and M2-related genes (i.e., *Spp1* and *Cxcl3*) along pseudotime, the region in shade indicates the 95% confidence intervals. **i** Scatter plot shows the correlation of proportions of *SPP1*^+^ TAMs and *CD74*^+^ TAMs in two CRC cohorts with public scRNA-seq data. The left plot includes 50 tumor samples from the GSE178341 dataset, while the right plot includes 15 tumor samples from the GSE132465 dataset. **j** Representative spatial feature plots show the signature score of Immune cells, *CD74*^+^ TAMs and *SPP1*^+^ TAMs in tumor sections from a CRC patient sample (Qi et al.^51^). **k** Scatter plot reveals the correlation between signature scores of *CD74*^+^ TAMs and *SPP1*^+^ TAMs in immune cell-enriched region of the CRC tumor sample in (**j**). **l** Kaplan–Meier survival curves demonstrate the correlation of tumor-infiltrating *CD74*^+^ TAM/*SPP1*^+^ TAM proportion ratio with ICI treatment outcomes in two immunotherapy cohorts. **m** Box-and-whisker plots showing changes in the proportion of specific TAMs subtypes before and after treatment in the FMT cohort. The left panel represents the proportion of *SPP1*⁺ TAMs, the middle panel represents *CD74*⁺ TAMs, and the right panel shows the difference between *CD74*⁺ TAMs and *SPP1*⁺ TAMs. NR (non-responders) and R (responders) indicate different response groups to the treatment. The dataset includes a total of nine pairs of samples. **n** Bar plots illustrating the expression levels of macrophage-associated genes in M1 and M2 macrophages cultured under *Akk*-conditioned medium (*Akk*-CM) vs. control medium (Ctrl-Medium). The top row shows *Cd74*, *Il6*, and *Inos* expression in M1 macrophages, while the bottom row represents *Cd163*, *Cd206*, and *Spp1* expression in M2 macrophages. Statistical significance is conducted with three replicates and indicated as *p < 0.05, **p < 0.01, and ***p < 0.001

Given the well-established role of macrophage M1/M2 polarization in tumor,^46,47^ we found that both macrophages and monocytes in the PW group exhibited M1 polarization, whereas they displayed M2 polarization in the PA group (Supplementary Fig. 7a, b), Moreover, DEGs between the PW and PA groups were enriched in pathways related to macrophage polarization (e.g., IL1, IL10, IFN-γ signaling, and NF-κB activation) and the “response to bacterial associated molecule” pathway (Supplementary Fig. 7c). By deconvoluting M1 and M2 gene sets in the two immunotherapy cohorts with available follow-up information, we observed a significant association of the M1/M2 ratio with immunotherapy prognosis (Fig. 4a and Supplementary Fig. 7d, e). These findings support the crucial role of gut microbiota in maintaining pro-inflammatory tumor-infiltrating macrophages.^20^ We next investigated the heterogeneity of tumor-infiltrating myeloid cells among the four groups, identifying seven subtypes based on specific markers (Fig. 4b, c). The proportions of cDC and *Cd74*^+^ macrophages, which act as antigen-presenting cells (APCs) through high expression of *Cd74*, were higher in the PW group, whereas the proportion of *Spp1*^+^ macrophage was reduced (Fig. 4d and Supplementary Fig. 7f). Similar trends were observed in normobiotic vs. germ-free mice (Fig. 4e), suggesting the contribution of gut microbiota to macrophage reprogramming. To further explore this, we performed trajectory inference analysis and identified seven states along the evolutionary paths from the pseudotime start timepoint (Fig. 4f). Notably, *Spp1*^+^ TAMs and *Cd74*^+^ TAMs were enriched in distinct trajectory paths (i.e., T1 and T2) and states (i.e., *Spp1*^+^ TAMs in state 4 and *Cd74*^+^ TAMs in state 7) (Fig. 4f, g). As *Spp1*^+^ TAMs and *Cd74*^+^ TAMs exhibited the highest M2 and M1 scores respectively (Supplementary Fig. 7g), the expression of *Cd74* and MHC-II component genes (e.g., *H2-Dma*) increased along the T1 trajectory path during evolution but decreased along the T2 path In contrast, expression of *Spp1* and M2 polarization-related chemokine (e.g., *Cxcl3*)^48^ showed the opposite direction (Fig. 4h).

Using public scRNA-seq data of patients with colorectal cancer,^49,50^ we observed a significant negative correlation between the proportions of *SPP1*^+^ TAMs and *CD74*^+^ TAMs (Fig. 4i and Supplementary Fig. 7g–k), further supporting the distinct evolutionary paths of inflammatory (i.e., *SPP1*^+^) and antigen-presenting (i.e., *CD74*^+^) TAMs. We further validated this potential reprogramming using spatial transcriptomic profiles of primary tumor samples from colorectal cancer patients, consistently observed significant negative correlations between *SPP1* and *CD74* expression within immune cells-enriched region (e.g., tertiary lymphoid structures) (Fig. 4j, k, and Supplementary Fig. 8a–e)^51^. Through CIBERSORT-based deconvolution of public bulk transcriptome data from two immunotherapy cohorts, deconvoluted *SPP1*^+^ TAM proportion significantly correlated with poor prognosis, whereas deconvoluted *CD74*^+^ TAMs and the ratio of *CD74*^+^/*SPP1*^+^ TAMs significantly correlated with favorable prognosis (Fig.4l and Supplementary Fig. 8f–j). Additionally, we estimated shifts in these two TAM subtypes using public data from FMT clinical trials, where fecal samples from FMT donors who achieved complete remission after PD-1 inhibitor monotherapy were transferred to ten refractory patients^15^. By comparing three responders and seven non-responders, we observed decreased deconvoluted *SPP1*^+^ TAM and increased *CD74*^+^ TAM proportions post-FMT in responders compared to non-responders, resulting in a significant shift of *SPP1*^+^ TAMs to *CD74*^+^ TAMs (Fig. 4m). Experimentally, we induced the polarization of mouse bone marrow-derived macrophages into M1 and M2 phenotypes. After adding conditional medium of specific bacteria species (i.e., *Akkermansia muciniphila*, referred to as *Akk*^11^) known to enhance ICI efficacy, the expression of M1 marker genes (e.g., *Inos* and *Il6*) and *Cd74* was significantly increased, while the expression of M2 marker genes (*Cd163* and *Cd206*) and Spp1 was significantly decreased (Fig. 4n and Supplementary Fig. 8k). These results suggested that microbiota may induce potential microphage reprogramming from M2 polarization-related TAMs (e.g., *Spp1*^+^ TAMs) to APCs (e.g., *Cd74*^+^ TAMs), potentially enhancing immunotherapy efficacy.

### Effects of SPP1 on the Efficacy of ICI treatment

**Fig. 5:**
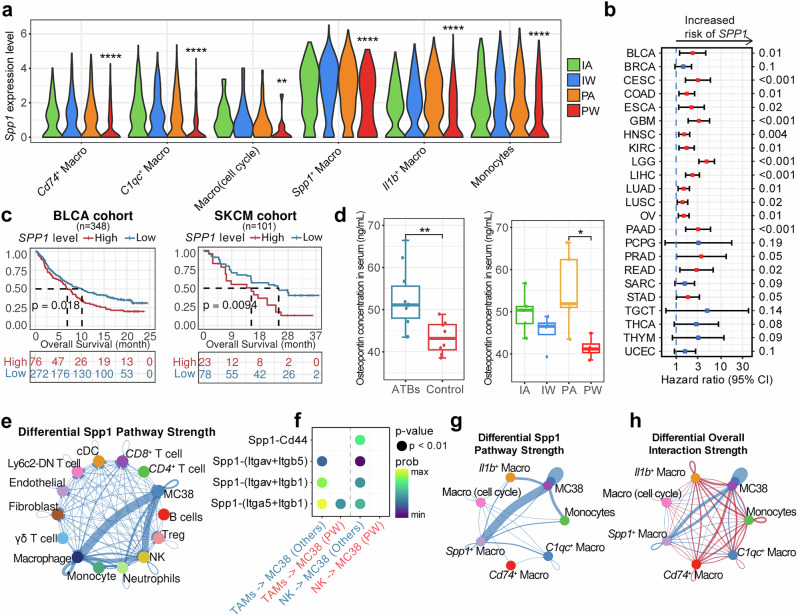
Synergistic effects of gut microbiota and ICI treatment on *SPP1.* **a** Violin plots reveal *Spp1* expression levels in myeloid subtypes across four groups. **b** Forest plot reveals the association between *SPP1* gene expression levels and the prognosis of major cancer types in the TCGA dataset. **c** Kaplan–Meier survival curves show the correlation between *SPP1* expression levels and prognosis in two immunotherapy cohorts. **d** Box plots indicate the levels of osteopontin measured by ELISA in the serum of mice across four groups. Each group consists of 4 mice. **e** Network diagram illustrates the differential interaction strength of the Spp1-related pathway between two cell types in the PW group compared to the others. **f** Bubble plot displays the interaction strength of the Spp1-related pathways between tumor-associated macrophages (TAMs)/NK cells and MC38 tumor cells in the PW group compared to the others. **g** Network diagram shows the differential interaction strength of the Spp1-related pathway between myeloid subtypes and MC38 cells in the PW group compared to the others. **h** Network diagram illustrates the differential interaction strength of the overall signaling between myeloid subtypes and MC38 cells in the PW group compared to the others

Besides the alteration in the proportion of *Spp1*^+^ TAM, *Spp1* expression was ubiquitous across different cell types at varying levels and is consistently reduced in the PW group (Fig. 5a), suggesting the potential role of *Spp1* expression in the synergistic interaction between gut microbiota and ICI treatment. Interestingly, higher *SPP1* expression consistently correlated with poor prognosis across multiple cancer types from The Cancer Genome Atlas (TCGA) project (Fig. 5b), as well as the two immunotherapy cohorts (Fig. 5c). Moreover, lower levels of ELISA-estimated osteopontin, a secreted phosphoprotein encoded by *Spp1*, were observed in the serum of mice with intact gut microbiota, particularly when combined with ICI treatment in the PW group (Fig. 5d). We further compared the differential *Spp1*-related interaction intensity between cancer cells (i.e., MC38) and all TME cell types among four treatment groups, identifying the greatest decrease in the PW group, particularly in TAMs and especially *Spp1*^+^ TAMs (Fig. 5e–g). Indeed, only the *Spp1*^+^ TAM subtype exhibited decreased interactions with MC38 compared to other myeloid subtypes (Fig. 5h). These findings suggest that gut microbiota may inhibit tumor progression and enhance the immunotherapy efficacy by downregulating *Spp1* expression, with osteopontin potentially serving as a prognosis marker in tumor progression and treatment outcomes.

**Fig. 6:**
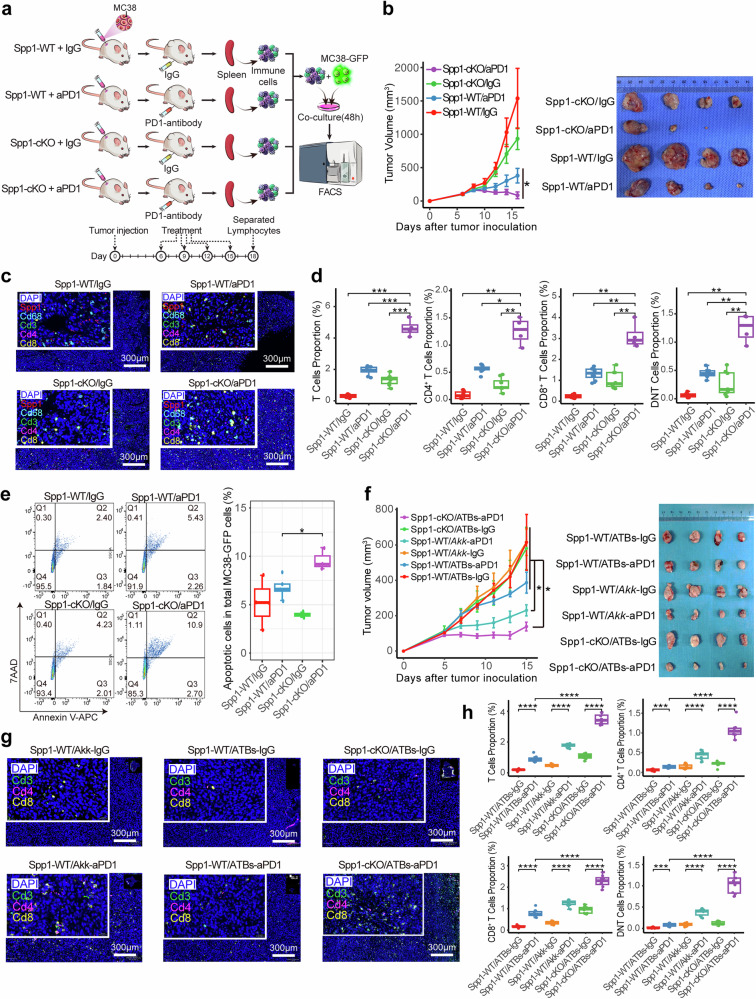
Effects of Spp1 depletion in macrophage on ICI treatment in mouse model. **a** Schematic representation of the experimental design with ICI/IgG treatment in *Spp1*-cKO mice compared to *Spp1*-WT mice. **b** Line graph (left) depicts the volumetric progression of subcutaneous tumors across four mouse groups from the study depicted in (**a**). Two batches of experiments were performed with four mice in each group. The image (right) shows the comparison of excised tumors among four groups for one batch. Data are represented as mean ± SEM. **c** Representation of multiplex immunofluorescence (mIF) images display the staining of T cells and *Spp1*^+^ macrophages within the subcutaneous tumors across four groups as shown in (**b**). **d** Bar plot quantifies the mIF-based proportions of T cell subtypes within the subcutaneous tumors of the four mouse groups, as shown in (**b**). Each group comprises four tumor samples except the Spp-cKO/aPD1 group (*n* = 2 available for mIF), with data collected from two sections per tumor. **e** Paired cell flow cytometry panel (left) and box plot (right) depict the percentage of apoptotic MC38 cells co-cultured with the spleen-derived T cells across four groups. Each group consists of four mice. **f** Line graph (left) depicts the volumetric progression of subcutaneous tumors across six ATBs-treated mouse groups with ICI/IgG treatment in *Spp1*-cKO mice compared to *Spp1*-WT mice. The image (right) shows the comparison of excised tumors among six groups. Each group consists of four mice. Data are represented as mean ± SEM. **g** Representation of mIF images reveals the spatial distribution of T cells within the subcutaneous tumors across six groups, as shown in (**f**). **h** Bar plot demonstrates the proportions of T cell subtypes in subcutaneous tumors across six mouse groups, as shown in (**f**). Each group comprises four tumor samples, with data collected from two sections per tumor sample

Given the role of *SPP1*^+^ TAMs in tumor malignancy, progression and prognosis highlighted in previous studies,^32,52–54^ we explored their role in ICI treatment in the context of gut microbiota using Spp1^flox/flox^Lyz2-Cre^+^ conditional knockout mice (referred to as Spp1-cKO), in which *Spp1* is specifically deleted in myeloid cells (e.g., macrophages) (Supplementary Fig. 9a). MC38 cells were subcutaneously implanted into Spp1-cKO and Spp1^flox/flox^Lyz2-Cre^−^ (refer as to Spp1-WT), followed by ICI treatment and subsequent immune cell sorting for co-culture assay (Fig. 6a). One-day delayed ICI treatment after subcutaneous implantation of MC38 cells resulted in larger initial tumor volume and slower growth rate but did not reduce tumor volume (Figs. 1b and 6b). More importantly, Spp1-cKO mice exhibited significantly reduced tumor growth and better ICI treatment efficacy based on tumor volume (Fig. 6b), paralleled by an increase in tumor-infiltrating *CD4*^+^/*CD8*^+^ T cells and DNT cells (Fig. 6c, d and Supplementary Fig. 9b). Consistently, co-culture with splenic lymphocytes from ICI-treated Spp1-cKO mice induced a significantly higher apoptotic rate of GFP labeled MC38 cells, as measured by flow cytometry (Fig. 6e), suggesting that an *Spp1*^+^ macrophage-free TME may enhance the tumoricidal activity of anti-PD-1 treatment by stimulating more cytotoxic T cells.

As the efficacy of ICI-mediated immunotherapy decreases in the PA group mice after gut microbiota clearance, this effect can be restored by oral supplementation with specific functional bacteria species (i.e., *Akk*^11^) and re-established gut microbiome (Fig. 6f and Supplementary Fig. 9c), we next investigated the impact of *Spp1*^+^ TAM depletion on ICI efficacy in ATB-treated mice. Notably, Spp1-cKO mice still responded to anti-PD-1 treatment after gut microbiota clearance, in contrast to the loss of efficacy observed in Spp1-WT mice (Fig. 6f). Accordingly, posted-treatment tumors from Spp1-cKO mice exhibited significantly higher proportions of various types of infiltrating T cells (Fig. 6g, h and Supplementary Fig. 9d). As validation, oral supplementation with *Faecalibacterium prausnitzii* (FP), another recently reported bacteria species that enhances ICI sensitivity^55^, in ATB-treated mice also increased the infiltrating T cells and restore the efficacy of PD-1 inhibitor (Supplementary Fig. 10). These findings suggest that *Spp1*^+^ TAM in the TME may play an important role in gut microbiota-modulated ICI-based immunotherapy.

### γδ T cell-APC-*CD8*^+^ T cell axis mediated *CD8*^+^ T stimulation via NF-κB pathway

**Fig. 7:**
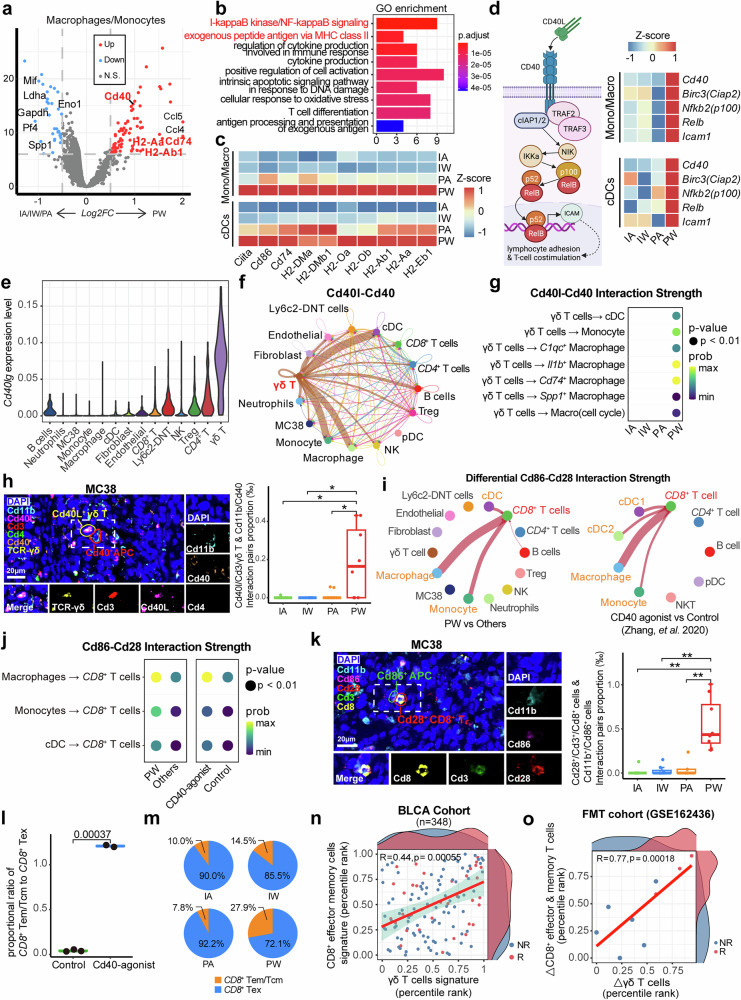
Stimulation of *CD8*^+^ T by APCs mediated by γδ T cells. **a** Volcano plot delineates the differentially expressed genes (DEGs) in macrophages and monocytes from subcutaneous tumors of the PW group compared with those from the others. **b** Bar graph shows the pathway enrichment analysis of DEGs as shown in (**a**). **c** Heatmap presents the expression levels of MHC-II-related genes in antigen-presenting conventional dendritic cells (cDCs) and monocytes/macrophages across four groups. **d** Schematic graph (left) depicts the key genes in the non-canonical NFκB pathway, the expression of which in cDCs and monocyte/macrophages was illustrated in the heatmap (right) across four mouse groups. Created with BioRender.com. **e** Violin plot reveals the MAGIC^91^-imputed expression levels of *Cd40lg* across all cell types. **f** Network graph portrays the cell interaction strength between different cell types within the Cd40l-Cd40 pathway. **g** Bubble plot indicates the interaction strength of the Cd40l-Cd40 pathway between γδ T cells and cDCs, monocytes, and macrophage subsets across four groups. **h** Representative multiplex immunofluorescence (mIF) images (left) illustrate the spatial distribution of interacting Cd40l^+^ γδ T cells and Cd40^+^ APCs within the subcutaneous tumors, box plot (right) shows the numbers of interacting Cd40l^+^ γδ T cells and Cd40^+^ APC pairs within the subcutaneous tumors across four groups. Each group comprises four tumor samples, with data collected from two sections per tumor. **i** Network graphs on the left show the differential interaction strength of the Cd86-Cd28 pathway between tumor-infiltrating *CD8*^+^ T cells with other cell types in the PW group compared with those in the other three groups; network graphs on the right display the differential interaction strength of the Cd86-Cd28 pathway between tumor-infiltrating *CD8*^+^ T cells in Cd40 agonist-treated mice compared to those in control mice. **j** Bubble plot demonstrates the interaction strength of the Cd86-Cd28 pathway between tumor-infiltrating macrophages/monocytes/cDCs and *CD8*^+^ T cells as shown in (**i**). **k** Representative mIF images (left) illustrates the spatial distribution of interacting Cd86^+^ APC and Cd28^+^Cd8^+^ T cells within the subcutaneous tumors, box plot (right) shows the numbers of interacting Cd86^+^ APC and Cd28^+^Cd8^+^ T cells pair within the subcutaneous tumors across four groups. Each group comprises four tumor samples, with data collected from two sections per tumor. **l** Box plot compares the ratio of tumor-infiltrating *CD8*^+^ effector memory/central memory T cells to *CD8*^+^ exhausted T cells between Cd40 agonist-treated mice (*n* = 2) and control groups (*n* = 3). **m** Pie chart illustrates the ratio of *CD8*^+^ effector memory/central memory T cells proportion to *CD8*^+^ exhausted T cells across four groups. **n** Marginal density scatter plot shows the proportion of tumor-infiltrating *CD8*^+^ effector memory and γδ T cells in patients from BLCA immunotherapy cohorts. **o** Scatter plot illustrating the linear correlation between changes in γδ T cell abundance and *CD8*^+^ effector & memory T cell abundance before and after FMT. The *x*-axis represents the change in γδ T cells, while the *y*-axis represents the change in *CD8*^+^ effector & memory T cells, both expressed as percentile rank changes. Density distributions of NR (non-responders, blue) and R (responders, red) groups are shown on the top and right sides of the plot. The dataset includes a total of nine pairs of samples

Similar to DCs, specific macrophage subtypes can also act as APCs, impacting immunotherapy efficacy by promoting the differentiation of cytotoxic T lymphocytes and preventing their dysfunction through specific interactions.^56,57^ Through scRNA-seq analysis, we found up-regulation of Cd40 and several MHC-II components (e.g., H2-Ab1 and Cd74) in both macrophages/monocytes and DCs from the PW group (Fig. 7a and Supplementary Fig. 11a). Consistently, DEGs in the PW group were enriched in multiple antigen presentation-related pathways, including “exogenous antigen via MHC-II” (Fig. 7b and Supplementary Fig. 11b). Components of these pathways were significantly upregulated in APCs from the PW group, particularly in *Cd74*^+^ TAM (Fig. 7c and Supplementary Fig. 11c, d). These findings suggested that gut bacteria and PD-1-based immunotherapy may synergistically enhance the antigen presentation capability of APCs, subsequently inducing the specific activation of *CD4*^+^-Tem cells in the PW group.

On the other hand, I-κB/NF-κB regulation signaling was also enriched in APCs from the PW group (Fig. 7b and Supplementary Fig. 11b, e), with key genes specifically upregulated, including *Cd40*, *Birc3* (*Ciap2*), *Nfkb2* (*p100*), *Relb*, and *Icam1* (Fig. 7d). This finding is consistent with previous reports indicating that the presence of bacteria (including gut microbiota) can induce up-regulation of Cd40 to activate APC cells through the NF-κB pathway.^20,58,59^ Additionally, the expression of Il12b, which is produced by non-classical NF-κB pathway activated DCs and enhance anti-PD-1 therapy,^56^ was also upregulated in APCs (Supplementary Fig. 11f, g). Given that the interaction between CD40 and CD40L (encoded by *CD40LG*) is crucial for APC regulation and NF-κB pathway-based activation,^56^ we next estimated the *Cd40lg* expression in different cell types. Consistent with the crosstalk between APCs and tumor-infiltrating *CD4*^+^ T and B cells,^59,60^ *Cd40lg* exhibited high expression in these two TME components (Fig. 7e). Interestingly, the highest expression of *Cd40lg* was observed in γδ T cells (Fig. 7e), while the IA group showed significantly lower *Cd40lg* expression than other groups in γδ T cells but not in B cells or *CD4*^+^ T cells (Supplementary Fig. 12a), suggesting that both intact gut microbiota and ICI may enhance *Cd40lg* expression specifically in γδ T cells. Consistently, the Cd40l-Cd40 interaction signal was enriched between γδ T cells and APCs according to Cellchat-based cell-cell communication analysis (Fig. 7f), and the Cd40lg-Cd40 ligand/receptor pair exhibited the highest interaction strength in the PW group, particularly between γδ T cells and *Cd74*^+^ TAMs (Fig. 7g).

**Fig. 8:**
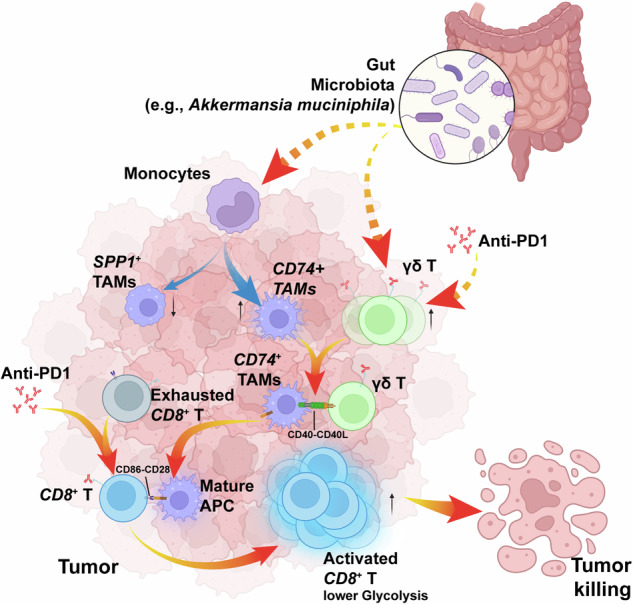
Schematic summary of the synergistic effect of gut microbiota and ICI treatment on macrophage reprogramming and T cell activation *via* γδT cell-APC-*CD8*^+^ T cell axis. TAM tumor-associated macrophage, APC antigen-presenting cells. Created with BioRender.com

Through experimental validation using mIF, the number of interacting Cd40L^+^ γδ T cells and Cd40^+^ APCs pairs were significantly higher in the PW group compared to the other three groups in both MC38- and CT26-derived tumors (Fig. 7h and Supplementary Fig. 12b), whereas interacting Cd40l^+^Cd4^+^ T cells and Cd40^+^ APC pairs are almost absent in all groups (Supplementary Fig. 12c). Given that APCs can activate *CD8*^+^ T cells through CD86-CD28 interactions,^61^ which exhibited the strongest strength in the PW group (Fig. 7i), we further investigated whether activating Cd40 could enhance adaptive immunity. After experimentally activating CD40 with CD40 agonist,^62^ we observed the activation of *CD8*^+^ T cells by APCs, particularly by macrophages, through increased interaction strength of the Cd86-Cd28 co-stimulatory receptor pair, similar to the effect of gut bacteria (Fig. 7i, j). Through mIF assay, significantly more prolific interactions between Cd28^+^Cd8^+^ T cells and Cd86^+^ APCs were observed in the PW group of both MC38- and CT26-derived tumors (Fig. 7k and Supplementary Fig. 12d). Subsequently, the ratio of tumor-infiltrating *CD8*^+^ central/effect memory T cells vs. exhausted *CD8*^+^ T cells was significantly increased after CD40 agonist treatment (Fig. 7l), consistent with observation in the PW group (Figs. 7m and 2d). Moreover, significant correlations were observed between deconvoluted scores of *CD8*^+^ effect memory T and γδ T cell signatures in three immunotherapy cohorts, with scores of both signatures being higher in responders than in non-responders (Fig. 7n and Supplementary Fig. 12e, f), thus contributing to their prognostic value of immunotherapy (Fig. 3f, and Supplementary Fig. 3d). The same trend was also observed using the data from the clinical FMT trial described above,^15^ revealing significantly positive correlation between dynamic infiltrating γδ T cell proportions and those of *CD8*^+^ Tem post-FMT treatment, which were significantly enriched in responders (Fig. 7o and Supplementary Fig. 12g). Our findings suggested that gut bacteria may synergistically improve the efficacy of immunotherapy through a potential Cd40l-Cd40 mediated γδ T cell-APC-*CD8*^+^ T cell axis (Fig. 8).

## Discussion

Extensive studies have underscored the influence of gut microbiota on the efficacy of ICI-based immunotherapy through metabolites and ligand-receptor interactions (e.g., PD-L2-RGMb).^11,17,19,21^ With the advent of scRNA-seq technologies, understanding of cell proportion changes and molecular signals in the TME has deepened,^23^ including the regulatory role of gut microbiota in monocyte reprogramming.^20^ However, a systematic investigation at the single-cell level of gut microbiota’s impact on TME post-ICI treatment was lacking. In our study, we performed scRNA-seq and subsequent validations (e.g., flow cytometry and mIF) in mouse models to evaluate the synergistic effects of gut microbiota and ICI on treatment outcomes by influencing specific TME components and their functions.

ICI treatment alone can increase *CD8*^+^ T cell proportions in the TME,^9^ while the combination of healthy human microbiota transplantation and ICI treatment can synergistically facilitate the accumulation of both *CD4*^+^ and *CD8*^+^ T cells in tumor-draining lymph nodes after ICI.^21^ Our findings further indicate that an intact gut microbiota, combined with ICI treatment, may substantially elevate the proportion of specific TILs (e.g., *CD4*^+^ T cells, *CD8*^+^ effector/central memory T cells, and γδ T cells). Although ICI-based immunotherapy has been reported to be capable of rejuvenating exhausted T cells,^63^ we highlighted the important role of gut bacteria in potentiating the ICI-mediated amelioration of *CD8*^+^ T cell exhaustion. Specifically, intact gut microbiota combined with ICI treatment in mice resulted in increased proportions of total tumor-infiltrating *CD8*^+^ T cells as well as the ratio of memory/effector to exhausted *CD8*^+^ T cells. Mechanistically, PD-1 promotes T cell exhaustion by targeting the co-stimulatory receptors CD28, which plays an indispensable role in T cell activation and PD-1-based immunotherapy.^63,64^ Given the activation of T cells via APCs through CD86-CD28 interactions, the elevation of such co-stimulatory signaling in the PW group suggested that gut microbiota may impact immunotherapy outcomes through ICI-driven reversal of *CD8*^+^ T cell exhaustion. Moreover, we revealed that gut microbiota can significantly reduce glycolytic levels in *CD8*^+^ T cells, consistent with the enhancement of *CD8*^+^ T cell memory and antitumor function after inhibiting glycolytic metabolism.^65^

The possible mechanism underlying the synergistic effect of gut microbiota and ICI treatment on T cell activation has recently been demonstrated, emphasizing the importance of PD-L2- RGMb interactions between DC and T cells. Blocking this interaction would increase the PD-L2-PD-1 binding, attenuate PD-1-induced T cell exhaustion, promote T cell activation and enhance their antitumor functions.^21^ On the other hand, microbiota may trigger STING-type I IFN-dependent monocyte reprogramming from monocytes to protumor macrophages,^20^ aligning with our observation, particularly after ICI treatment. Moreover, we identified several macrophage subtypes, including M2 polarization-related *Spp1*^+^ TAMs, which exhibit protumor characteristics and negative prognostic value for cancer treatment, including immunotherapy.^32,51,54^ Notably, microbiota-induced reprogramming from *Spp1*^+^ TAM to antigen-presenting *Cd74*^+^ TAM was observed, particularly with the synergistic effect of ICI treatment. On the other hand, the function of *Spp1*^+^ TAM may rely on the high *Spp1* expression, which has not only been suggested as a negative prognostic factor for multiple cancer types,^66^ but also considered a potential drug target for cancer treatment.^67,68^ Indeed, the role of *SPP1* has been mostly demonstrated in TAMs, and significant enrichment of *SPP1*^+^ TAMs within the TME is implicated in promoting tumor progression.^32,54,62^ Importantly, We observed the greatest reduction of Spp1-related interaction strength between macrophage and tumor cells in the PW group. Meanwhile, a recent study revealed that conditionally knocking out *Spp1* in macrophages resulted in slower tumor growth rate and increased efficacy of ICI treatment in mouse model of liver cancer,^25^ highlighting the important role of *Spp1*^+^ TAMs in immunotherapy response. Given that specific bacteria species (e.g., *Akkermansia muciniphila*) have been identified to functionally impact the ICI treatment outcomes,^11,69^ and gut microbiota can suppress the *Spp1* expression in damaged liver tissue,^70^ our investigation into conditional *Spp1* knockout in macrophage using ATBs treatment and rescue with oral supplementation of *Akk*, as well as the stimulation of conditional medium in macrophage reprogramming, suggests that the gut microbiota likely functions upstream in the regulatory cascade, and influence on ICI treatment efficacy depends on modulating *Spp1*^+^ TAMs and attenuating *Spp1* expression, consistent with the negative association of *Akk* abundance with M2/M1 ratio^20^. Moreover, as PD-1 inhibitor treatment can also alter the composition of gut microbiota, including increasing the *Akkermansiaceae* abundance, this feedback loop may further amplify the therapeutic effectiveness of the PD-1 inhibitors.

Furthermore, we screened well-established ligand-receptor interactions among different cell types and identified a potential alternative mechanism involving the γδ T cell-APC-*CD8*^+^ T cell axis. Although the population of γδ T cells is small, they exert crucial cytotoxic effects on cancer cells through both direct mechanisms (e.g., deploying the pore-forming molecule perforin and pro-apoptotic protease granzyme B) and indirect mechanisms (e.g., DC cell activation or αβ T cell response promotion), thus serving as a bridge between innate and adaptive immunity.^71^ Importantly, a series of studies have established the role of tumor-infiltrating γδ T cells in TME and treatment outcomes of immunotherapy, particularly in MHC-I deficient patients.^42,43,72–74^ On the other hand, commensal microbial metabolites can augment host γδ T cell proportions,^75^ consistent with our findings in the treatment-responsive group (i.e., the PW group). Therefore, our study suggests a possible context for the interaction between gut microbiota and ICI treatment response through γδ T cells. Besides the increased proportion of tumor-infiltrating γδ T cells in the PW group, higher expression of *Cd40lg* was observed in γδ T cells than other cell types, particularly in mice with intact gut microbiota or/and ICI treatment, while the expression of its receptor (i.e., *Cd40*) was specifically elevated in APCs, such as *Cd74*^+^ macrophages, which are upregulated through macrophage reprogramming from *Spp1*^+^ macrophage in the PW group. Given that APC activation *via* CD40-CD40L-mediated non-canonical NF-κB pathway is essential for the antitumor effects of ICI therapy, and the gut microbiota can impact the treatment outcome of anti-CD40 immunotherapy,^56,76^ the strong Cd40-Cd40l interaction and enrichment of non-canonical NF-κB pathway in the PW groups, as well as tumor-infiltrating APCs activation induced by CD40 agonists, suggest that gut microbiota may enhance the ICI treatment outcome *via* Cd40l-Cd40 mediated γδ T cell-APC activation and subsequent CD86-CD28 mediated APC-*CD8*^+^ T cell activation.

Several limitations of our study should be noticed. First, the underlying mechanisms by which gut microbiota contribute to macrophage reprogramming, downregulation of *Spp1* expression, and γδ T-APC-*CD8*^+^ T cell axis in ICI immunotherapy remain unexplored through functional experiments. For instance, although the role of gut microbiota in γδ T and *Spp1*^+^ TAM can be determined through depleting with TCRγδ antibody and conditional knockout mouse model, respectively, their impact on macrophage reprogramming from *Spp1*^+^ TAM toward *Cd74*^+^ TAM remains to be elucidated. Second, the number of single γδ T cells obtained in this study was relatively small, hindering detailed research into the behavior and role of γδ T cells in ICI-based immunotherapy. Finally, as a series of bacteria species are involved in the immunotherapy response, exploring the specific species involved in the synergistic effect through the underlying mechanisms we demonstrated is warranted in future studies.

In conclusion, we conducted scRNA-seq using tumor samples from 2 × 2 matrix mouse groups related to immunotherapy and identified the possible synergistic effects of gut microbiota and ICI on TME components and molecular signaling. Our results provided insights into the possible mechanisms by which gut microbiota impact the efficacy of ICI-based immunotherapy, including promoting macrophage reprogramming, regulating *Spp1* expression in the TME, and activating T cell through γδ T cell-APC-*CD8*^+^ T axis.

## Materials and methods

### Animal breeding and treatment

Female C57BL/6 mice, aged 6 to 8 weeks, were bred and maintained in a specific pathogen-free (SPF) animal facility. Gut microbiota depletion was achieved through the daily administration of a broad-spectrum antibiotic (ATBs), containing ampicillin (1 mg/ml), streptomycin (5 mg/ml), colistin (1 mg/ml), and vancomycin (0.25 mg/ml) sourced from Shanghai Yuan Ye Biotechnology. The antibiotics were added to the sterile drinking water of the mice, with both the solution and bottles replaced three times weekly. The efficacy of the antibiotic treatment was verified by monitoring fecal microbial genome concentrations. Mice received two weeks of antibiotic treatment prior to tumor implantation, which continued throughout the MC38 sarcoma model experiment.

CRISPR-Cas9-based *Spp1*^flox/flox^ conditional knockout and Lyz2-Cre mice on a C57BL/6J background were purchased from GemPharmatech (China). *Spp1*^flox/flox^Lyz2-Cre⁺ (*Spp1*-cKO) and *Spp1*^flox/flox^Lyz2-Cre⁻ (*Spp1*-WT) mice were generated by breeding these two strains. In *Spp1*-cKO mice, exons 2 through 8 (which encompass the entire coding region of the *Spp1* gene) are deleted specifically in myeloid cells (e.g., macrophages), while *Spp1*-WT mice served as controls. The same breeding and treatment protocols were applied to both the *Spp1*-cKO and *Spp1*-WT mice.

### Cell incubation, subcutaneous tumor formation, and single-cell suspension preparation

MC38 cells (ENH204-FP, Kerafast) and CT26 cells were cultured in DMEM supplemented with 10% FBS and 1% HEPES (1 mmol/L, pH 7.4) at 37 °C in a 5% CO_2_ incubator. Syngeneic C57BL/6J mice were subcutaneously inoculated with 2 × 10^6^ MC38 cells, while syngeneic BALB/c mice received a subcutaneous injection of 1 × 10^6^ CT26 cells. When tumors reached approximately 60–90 mm^3^ (after ~5 days) for MC38 or 80–120 mm^3^ (after ~6 days) for CT26, the mice were intraperitoneally injected with 200 µg/mouse of anti-PD-1 monoclonal antibody (clone RMP1-14, BioXcell) or an isotype control (clone 2A3, BioXcell) at 3-day intervals for a total of four doses. Tumor sizes were monitored every two days using calipers.

Subcutaneous MC38 tumors were carefully excised and immediately placed on ice. The excised tissues were washed in Hanks’ Balanced Salt Solution (HBSS) to remove residual blood and contaminants, then finely minced and sheared prior to enzymatic digestion. Digestion was performed at 37 °C for 1 h using a cocktail containing collagenase I (2 mg/ml; Gibco, catalog number 1710-0017), collagenase IV (1 mg/ml; Gibco, catalog number 1710-4019), and 0.25% pancreatic enzymes (Gibco, catalog number 25200-056). After digestion, the tissue lysate was filtered through a 40-µm cell strainer to remove undigested material and large cell clumps. The filtered cell suspension was centrifuged at 500×*g* for 5 min at 4 °C, and the resulting cell pellets were washed to remove residual enzymes and debris. The pellets were then resuspended and subjected to erythrocyte lysis using 10× RBC Lysis Buffer (Thermo Fisher Scientific, catalog number 00-4300-54). Next, the cells were resuspended in HBSS supplemented with 0.04% bovine serum albumin (BSA). Cell viability and concentration were assessed using the Counting Star platform (Aber Instruments Ltd.). Following this, the cells were pelleted again at 500×*g* for 5 min at 4 °C and stored at −80 °C until further analysis. Finally, the single-cell suspension was adjusted to a final concentration of approximately 5000 cells per milliliter. The preparation of the single-cell suspension was conducted by Novogene Co., Ltd.

### Single-cell sample preparation and single-cell sequencing

Single-cell samples were prepared in accordance with the protocol outlined in the Chromium Single Cell 3’ Reagents Kits v2 User Guide. Briefly, we employed the Chromium Single Cell 3’ Library & Gel Bead Kit v2 (PN-120237), the Chromium Single Cell 3’ Chip Kit v2 (PN-120236), and the Chromium i7 Multiplex Kit (PN-120262). The single-cell suspension was washed twice with Phosphate-Buffered Saline (PBS) supplemented with 0.04% BSA. Cell quantity and concentration were verified using the TC20 Automated Cell Counter. Gel Beads in Emulsion (GEMs) were generated using a 10x Genomics Chromium Controller. Barcoded complementary DNAs (cDNAs) were then prepared using the 10x Genomics Chromium Single Cell 3’ reagent kit (V2 chemistry) and subsequently purified and amplified for library construction.

The quality and concentration of the cDNA libraries were assessed using an Agilent Bioanalyzer 2100. Libraries that met quality control criteria were subjected to PE150 sequencing on Illumina’s NovaSeq 6000 platform. All these procedures were conducted by Novogene Co., Ltd.

### Single-cell transcriptome data analysis

Analysis on single-cell transcriptome was performed with the pipeline as we described previously.^31–34,77^ Briefly, the raw data were processed using Cell Ranger (v3.0) as per the default settings. A raw unique molecular identifier (UMI) count matrix was generated, which was then converted into a Seurat object using the R package Seurat.^78^ Low-quality cells, defined by cells with UMI numbers below 500, gene numbers below 200 or greater than 8000, or mitochondrial-derived UMI counts of more than 15%, were filtered out. Additionally, potential doublets were identified and removed using Scrublet (v0.2) with expected doublet rate = 0.06.^79^ Cells passed quality control were left for subsequent analysis.

Single-cell transcriptome analysis was performed using the pipeline described previously.^31–35^ Briefly, the raw data were processed with Cell Ranger (v3.0) using default settings.^80^ A raw unique molecular identifier (UMI) count matrix was generated and then converted into a Seurat object using the R package Seurat.^78^ Low-quality cells—defined as cells with UMI counts below 500, gene counts below 200 or above 8000, or mitochondrial-derived UMI counts exceeding 15%—were filtered out. Additionally, potential doublets were identified and removed using Scrublet (v0.2) with an expected doublet rate of 0.06.^79^ Cells that passed quality control were used for subsequent analysis.

### Data integration and normalization

Data integration was performed using the Seurat v4 data integration pipeline.^78^ After total-count normalization and log-transformation, the top 3000 highly variable genes across the samples were identified. Subsequently, SCTransform was employed for data scaling and to correct for mitochondrial read percentages.^81^ Non-biological batch effects were removed using the canonical correlation analysis implemented in the Seurat functions FindIntegrationAnchors and IntegrateData.

### Cell clustering and annotation

Following data integration, principal component analysis (PCA) was performed using the RunPCA function, and UMAP was generated using the RunUMAP function. Cells were clustered at a resolution of 2.0 using Seurat’s FindNeighbors and FindClusters functions. Cell type annotation was achieved by manually examining marker genes based on canonical marker expression.

### Analysis of correlation of *CD8*⁺ T cell subtypes

To characterize the distinct states of *CD8*⁺ T cells, we employed previously defined signature genes.^40^ Specifically, the top 50 differentially expressed genes (DEGs) with the lowest *p*-values were selected as signature genes for each *CD8*⁺ T cell state. Subsequently, signature scores for each cell type were calculated using the AddModuleScore function available in the Seurat package. These scores were used as metrics to assess the correlation between the signature genes and the distinct *CD8*⁺ T cell states.

### Trajectory analysis of *CD8*⁺ T cells

For the trajectory analysis, Monocle 2 was employed to conduct pseudotime analysis, thereby elucidating the developmental trajectory and transitional relationships among *CD8*⁺ T cell subtypes.^82^ Initially, a set of 2000 significantly variable features was selected using the FindVariableFeatures function in Seurat with the variance-stabilizing transformation (vst) method. These features were then used to order the cells along the trajectory. Subsequently, size factors and dispersions were calculated for each cell to normalize the data. Differentially expressed genes along the trajectory were identified using the differentialGeneTest function provided by Monocle 2. For dimensionality reduction, we adopted the “DDRTree” method to better represent the underlying structure of the data. Following cell ordering, trajectories were visualized using Monocle 2’s plot_cell_trajectory function.

### Definition of myeloid and T cell-related phenotypes

For the analysis of macrophages and dendritic cells (cDCs), specific phenotypic signatures were employed as outlined in this section. The M1 and M2 macrophage phenotypes were classified based on the mean expression of predefined gene signatures. The gene set for the M1 macrophage signature includes *Nos2*, *Ccr7*, *Tnf*, *Inhba*, *Il12b*, *Il6*, *Il1b*, and *Cd86*. The M2 macrophage signature includes genes such as *Fn1*, *Arg1*, *Chil3*, *Egr2*, *Mrc1*, and *Retnla*.^83^ The glycolysis signature in macrophages was determined using a gene set that includes *Hk1*, *Hk2*, *Hkdc1*, *Gpi1*, *Pfkl*, *Pfkm*, *Aldoa*, *Tpi1*, *Gapdh*, *Pgk1*, *Pgam1*, *Pgam2*, *Eno1*, *Eno2*, *Pklr*, and *Pkm*.^84^ For the classification of cDC phenotypes, we focused on three major aspects: maturation, migration, and regulatory functions. The maturation signature for cDCs includes *Cd40*, *Cd80*, *Cd86*, *Relb*, and *Cd83*. The migration signature comprises *Ccr7*, *Myo1g*, *Cxcl16*, *Adam8*, *Icam1*, *Fscn1*, *Marcks*, and *Marcksl1*. The regulatory signature consists of *Cd274*, *Pdcd1lg2*, *Cd200*, *Fas*, *Aldh1a2*, *Socs1*, and *Socs2*. All dendritic cell-related gene sets were based on the work of Maier et al.^85^

### Cell-cell interaction analysis

To assess the complexities of cell-cell communication among various cell types, we quantified ligand-receptor pairs using the CellChat R package.^86^ Normalized gene expression matrices, along with metadata containing cell type annotations, served as the input data. We employed the CellChatDB.mouse database and conducted the analysis using the package’s default parameters. The netVisual_diffInteraction function was used to visualize the differential interaction strength between specific groups of cells, designating particular cells as sources or targets.

### Differentially expressed gene (DEG) analysis

For the analysis of differentially expressed genes (DEGs) in single-cell RNA sequencing (scRNA-seq) data, we employed a comprehensive approach using the edgeR and limma packages in R. Initially, the calcNormFactors function from edgeR was used to normalize gene expression counts. Subsequently, the voom function from limma was applied to stabilize the relationship between the variance and the mean of the gene expression data, thereby making it suitable for linear modeling. Finally, a linear model was fitted to the transformed data using limma, and Bayesian statistics were employed to compute significance *p*-values for identifying DEGs.

### Gene set enrichment analysis

In our study, we conducted gene set enrichment analyses utilizing the clusterProfiler (v4.0) package in R, encompassing both Over-Representation Analysis (ORA) and Gene Set Enrichment Analysis (GSEA) approaches.^87^ For ORA, we analyzed a refined set of differentially expressed genes (DEGs) using gene sets retrieved from the MSigDB database, which includes resources such as Gene Ontology, KEGG, WIKIPATHWAYS, and REACTOME. The enrichment analysis was performed using the enricher function to identify significantly enriched gene sets, highlighting the predominant biological processes and pathways influenced by our DEGs. In the GSEA, all genes were ranked based on their log₂FoldChange values in descending order. This ranked list was analyzed using the GSEA function in the clusterProfiler package to discern pathways exhibiting significant, concordant expression differences between two groups.

### Inferring the proportion of immune cells in RNA-seq data

To quantify the proportion of tumor-infiltrating activated *CD4*⁺ memory T cells, γδ T cells, macrophages M1, and macrophages M2 in both the RNA‑seq BLCA cohort^36^ and the melanoma cohort^37^, we employed the RNA deconvolution algorithm CIBERSORT. The analyses were conducted according to the guidelines provided by the developers and utilized the LM22 signature gene file available on the CIBERSORT platform (https://cibersort.stanford.edu/).^88^ The algorithm was executed with 1000 permutations, and statistical significance was evaluated using a two-tailed *p*-value, with a threshold of <0.01 for every sample. For the assessment of the proportion of *CD8*⁺ effector memory T cells and *CD8*⁺ central memory T cells in the aforementioned RNA‑seq BLCA and melanoma cohorts, we utilized the computational algorithm ssGSEA.

### Spatial transcriptomics data analysis

The Spatial Transcriptomics (ST) dataset used in this study was obtained from Mendeley Data (10.17632/ys6j8bndby.2). Gene expression information for the ST slides was captured using the Visium Spatial platform by 10x Genomics, which employs spatially barcoded mRNA-binding oligonucleotides according to the standard protocol. Raw sequencing reads from the ST data were quality-checked and mapped using Space Ranger v1.1. The gene-spot matrices generated from both the ST and Visium samples were subsequently analyzed using the Seurat package (version 4.4.0) in R. Spots with fewer than 200 detected genes and genes with fewer than 10 read counts—or expressed in fewer than three spots—were excluded from the analysis. Normalization across spots was performed using the LogVMR function. Dimensionality reduction was carried out using Principal Component Analysis (PCA), utilizing the first 30 principal components, and clustering was performed at a resolution of 1.1. Signature scoring derived from ST signatures was executed using the AddModuleScore function with default parameters in Seurat. Spatial feature expression plots were generated using the SpatialFeaturePlot function in Seurat (version 4.4.0). The specific gene signatures analyzed included: *SPP1*^+^ Macrophages: *SPP1*, *CXCL3*, *INHBA*. *CD74*^+^ Macrophages: *SLC40A1*, *CD74*. Immune Score: *PTPRC*, *BLK*, *CD19*, *TNFRSF13C*, *CD48*, *FDCSP*, *RHOH*, *MAP4K1*, *IKZF3*, *LTB*, *CD79B*, *IL7R*, *CD37*, *CD3E*, *TRBC1*, *TRBC2*, *TRAF3IP3*, *TRAC*, *IKZF1*, *FCMR*, *CD79A*, *RAC2*, *CD27*, *GPR183*, *IL16*.

### Flow cytometry

At 16 days post-inoculation, tumors, mesenteric lymph nodes (mLNs), blood, and spleens were harvested. Tumors were minced and digested in 5 ml PBS containing 1 mg/ml type IV collagenase (Sigma) at 37 °C for 1 h. Mesenteric lymph nodes and spleens were mechanically disrupted in RPMI medium. Following filtration through a 70 µm strainer (Corning), samples were centrifuged at 600×*g* for 5 min at 4 °C. Each sample was then incubated for 15 min at room temperature with Fixable Viability Stain 780 (565388, BioLegend; 1:1000 dilution). Subsequently, cell surface antigens were stained for 30 min at 4 °C using a fluorophore-conjugated antibody cocktail, including anti-mouse CD45 (103157, BioLegend), CD3ε (100353, BioLegend), CD8a (100728, BioLegend), CD279 (1109111, BioLegend), F4/80 (749284, BD), and CD11b (561114, BD). After surface staining, cells were fixed with the Fixation/Permeabilization Kit (554714, BD) prior to intracellular staining with anti-mouse CD206 (141729, BioLegend) and IFN-γ (505838, BD) for 30 min at 4 °C. Finally, cells were resuspended in 300 µl PBS and analyzed on a Symphony A5 flow cytometer (BD). Data were processed using FlowJo software.

### Immunohistochemistry

Freshly harvested tissues were fixed in 4% paraformaldehyde for 24 h, followed by graded dehydration, xylene treatment, and paraffin embedding. Tissue sections (4 µm) were baked at 65 °C for 2 h. Antigen retrieval was performed by heating the sections in a pressure cooker at 95 °C for 20 min using a retrieval buffer (pH 9.0 or pH 6.0). The sections, delineated with a hydrophobic barrier (Pap pen), were incubated with 3% H_2_O_2_ for 15 min to quench endogenous peroxidase activity, followed by overnight incubation at 4 °C with primary antibodies against CD3ε, CD4, and CD8α (Cell Signaling Technology; 1:100 dilution). Next, the sections were incubated for 30 min with a horseradish peroxidase-labeled goat anti-rabbit Ig mixture, then subjected to DAB staining (DAB-2031, MXB Biotechnologies), counterstained with hematoxylin, and finally examined under a microscope.

### Multiplex immunofluorescence staining

Using the Opal™ 7-Color Kit (Akoya Bioscience, NEL801001KT), tissues were sectioned into 4 µm slices and subjected to heat-induced antigen retrieval in either a citric acid or EDTA retrieval solution. The following primary antibodies were used: CD3ε (99940, Cell Signaling Technology, 1:100), CD4 (25229, Cell Signaling Technology, 1:100), CD8α (98941, Cell Signaling Technology, 1:100), CD68 (ab283654, Abcam, 1:500), SPP1 (ab218237, Abcam, 1:500), CD86 (19589, Cell Signaling Technology, 1:100), CD28 (ab243228, Abcam, 1:1000), CD11b (ab133357, Abcam, 1:1000), CD40 (ab252428, Abcam, 1:500), CD40L (ab65854, Abcam, 1:500), and TCR γ/δ (118101, Biolegend, 1:100). Pre-treatment with xylene and ethanol was performed prior to antigen retrieval, which was carried out for 20 min in a microwave at high power. Blocking was conducted using antibody diluent/block (Akoya Bioscience, 72424205) for 10 min, followed by primary antibody incubation for 1 h at room temperature or overnight at 4 °C. Secondary reagents and tyramide signal amplification (Opal series, 1:200, 10 min) were applied sequentially, with microwave-assisted antigen retrieval used for all markers. Finally, DAPI nuclear staining (5 min at room temperature) and application of anti-fade fluorescence mounting medium (ab104135, Abcam) preceded scanning with the Vectra Polaris system and analysis using QuPath software.

### Statistical analysis of cell interaction pairs proportion

We conducted statistical analysis on the staining results obtained from multiplex immunofluorescence staining to determine the proportion of cell interaction pairs. For each stained section, two regions were randomly selected for analysis, excluding areas such as tumor necrosis, image artifacts, and the section edges to avoid “edge effects”. Cell identification and counting were performed using QuPath software. Cells were annotated as either antibody-positive or antibody-negative based on the intensity of fluorescence labeling, and the software was used to measure the distance between cells. Directly interacting cells were defined as cell pairs with a physical distance of less than 15 μm. The proportion of cell interaction pairs of interest was calculated by dividing the total number of these cell pairs within the region by the total number of cells in that region.

### Bacterial culture

*Akkermansia muciniphila* (referred to as “*Akk*”) strains were acquired from the American Type Culture Collection (ATCC) and initially suspended in Brain Heart Infusion Broth (BHI) for culture. After suspension, the bacteria were evenly spread onto TSA blood agar plates containing 5% sheep blood and then incubated at 37 °C in an anaerobic chamber filled with 100% nitrogen. The media used—BHI (M330-01) and TSA blood agar plates (M206-03)—were both sourced from ELITE-MEDIA, USA.

*Faecalibacterium prausnitzii* (referred to as “*FP*”) strains were cultured under similar anaerobic conditions at 37 °C in a pre-reduced medium. For routine maintenance passaging, 30 µl of a late-log-phase *FP* culture was transferred into 5 ml of fresh pre-reduced medium in a 15 ml conical tube. For larger volumes (e.g., for in vivo experiments), 2 ml from the previous passage was inoculated into 50 ml of the same pre-reduced medium. Cultures were typically incubated for approximately 12 h, although the first passage following recovery from storage may require a longer incubation time to reach sufficient density.

### Gut microbiota supplementation experiment

One week prior to the experiment, the gut microbiota of the experimental mice was depleted by administering an antibiotic cocktail in the drinking water. The cocktail consisted of ampicillin (1 mg/ml), streptomycin (5 mg/ml), polymyxin (1 mg/ml), and vancomycin (0.25 mg/ml). Fecal samples were collected—both at the initiation and after two weeks of antibiotic administration (~100 mg per mouse)—to verify microbiota depletion by measuring DNA concentration. DNA was extracted from these samples using the Stool DNA Isolation Kit (FOREGENE) according to the manufacturer’s instructions. Subsequently, antibiotic treatment was discontinued.

Akkermansia muciniphila cultures grown on TSA blood agar plates were then collected and concentrated to 2 × 10^8^ CFU/200 μl in liquid medium and administered via oral gavage on a daily basis. In parallel, a separate group of mice received daily oral gavage of *Faecalibacterium prausnitzii* (*FP*) at the same concentration (2 × 10^8^ CFU/200 μl). On day 10, following the start of either *Akk* or *FP* supplementation, MC38 tumor cells were inoculated subcutaneously, and anti-PD-1 treatment was administered every two days for a total of four doses. Oral gavage (with either *Akk* or *FP*) continued throughout the duration of the experiment.

### 16S rRNA gene amplification and sequencing

Fecal genomic DNA was extracted using the CTAB/SDS method. The diluted DNA served as the template for amplifying the V3–V4 hypervariable regions of the 16S rRNA gene with barcoded primers. PCR reactions were performed using Phusion® High-Fidelity PCR Master Mix (New England Biolabs). Amplicons were pooled in equal concentrations and purified using the Qiagen Gel Extraction Kit (Qiagen, Germany). Libraries were then prepared with the TruSeq® DNA PCR-Free Sample Preparation Kit (Illumina, USA) according to the manufacturer’s instructions, and index codes were added. Library quality was assessed using a Qubit® 2.0 Fluorometer (Thermo Scientific) and an Agilent Bioanalyzer 2100. Finally, the libraries were sequenced on an Illumina NovaSeq 6000 platform to generate 250 bp paired-end reads.

### 16S rRNA amplicon analysis

All effective tags from each sample were clustered using the Uparse algorithm^89^ at a 97% sequence identity threshold. Multiple sequence alignment was performed with MUSCLE to generate a phylogeny of representative OTU sequences, which was subsequently used to calculate Bray–Curtis distances. A linear correlation analysis was conducted between the standard deviation (STDEV) of each compound’s β diversity distance and the corresponding number of hits; correlation coefficients (R) and *p*-values were then calculated. The R packages “microbiome” and “phyloseq” were employed to analyze and visualize changes at the OTU level across experimental groups.

### Isolation and polarization of bone marrow–derived macrophages

Bone marrow–derived macrophages (BMDMs) were prepared from mouse femurs. Briefly, femurs were dissected, and all attached muscle tissue was removed. After trimming away the epiphyses, the marrow cavities were repeatedly flushed with RPMI-1640 medium supplemented with 1% fetal bovine serum (FBS) to collect bone marrow cells. The resulting cell suspension was filtered through a 40 µm cell strainer to remove debris and then centrifuged at 2000 rpm for 10 min. The pellet was resuspended in RPMI-1640 containing 10% FBS, 1% penicillin–streptomycin, and 20 ng/ml M-CSF (novoprotein, catalog no. CB34). Cells were plated in 10 cm culture dishes and incubated at 37 °C under 5% CO_2_ until adherent bone marrow–derived macrophages (M0) were obtained.

To induce polarization, the adherent macrophages were dissociated, counted, and seeded at 2 × 10^5^ cells per well in 24-well plates. After allowing the cells to re-adhere, M1 polarization was induced with 100 ng/mL lipopolysaccharide (LPS; Sigma, catalog no. L2880) plus 50 ng/ml interferon-gamma (IFN-γ; novoprotein, catalog no. C746), and M2 polarization was induced with 20 ng/ml interleukin-4 (IL-4; novoprotein, catalog no. CK74) plus 20 ng/ml interleukin-13 (IL-13; novoprotein, catalog no. CX57). In parallel, cells were cultured in either 10% *Akk*–conditioned medium (*Akk*-CM) or control medium. After 24 h of stimulation, total RNA was extracted from each treatment group for quantitative PCR. The following primers were used to assess gene expression: The following primers were used to assess gene expression: Cd163-F: 5’-CCCTCACGGCACTCTTGGTTTG-3’; Cd163-R: 5’-GTCGCTGAATCTGTCGTCGCTTC-3’; Cd206-F: 5’-GGACGAAAGGCGGGATGTGTTG-3’; Cd206-R: 5’-GGGCTCTGGTGGGCGAGTC-3’; Spp1-F: 5’-ATCTCCTTGCGCCACAGAATGC-3’; Spp1-R: 5’-TCATCGTCATCATCGTCGTCCATG-3’; Cd74-F: 5’-GCCAGGAAGAAGTCAGCCACATC-3’; Cd74-R: 5’-GGGAACACACACCAGCAGTAGC-3’; Inos-F: 5’-GCAGGGAATCTTGGAGCGAGTTG-3’; Inos-R: 5’-TAGGTGAGGGCTTGGCTGAGTG-3’; Il6-F: 5’-AACGATGATGCACTTGCAGAAAAC-3’; Il6-R: 5’-TCTCTCTGAAGGACTCTGGCTTTG-3’. Gene expression levels were normalized to housekeeping controls and analyzed to evaluate the effects of each stimulus (control medium vs. *Akk*-CM) on M1 or M2 macrophage polarization.

### Co-culture experiment

Co-culture experiments were conducted as described previously.^90^ Briefly, mice were euthanized by cervical dislocation, and their spleens were harvested and placed on a 70 µm cell strainer set in a 6-well plate. Three milliliters of lymphocyte separation medium (Dakewe, DKW33-R0100) was added, and the spleens were minced with scissors before being gently ground using a syringe plunger. The resulting suspension was transferred to a 15 ml centrifuge tube and slowly overlaid with 1 ml of 1640 cell culture medium. The samples were then centrifuged at room temperature at 800×*g* (with acceleration and deceleration set at 2) for 30 min. The intermediate white membrane layer containing lymphocytes was carefully transferred to a new centrifuge tube and mixed with 10 ml of 1640 medium, followed by centrifugation at 1500 rpm for 3 min at room temperature. After discarding the supernatant, the cell pellet was washed twice with PBS, with each wash followed by centrifugation at 1500 rpm for 3 min. Cells were then counted and resuspended in PBS. One day prior to co-culturing, well-grown MC38-GFP cells were seeded in a 12-well plate at a density of 50,000 cells per well. Before co-culturing, the medium was replaced with 1 ml of fresh culture medium, and the previously isolated lymphocytes (50,000 per well) were added. After 48 h of co-culture, apoptosis of MC38-GFP cells was assessed using flow cytometry.

### Apoptosis detection via flow cytometry

The co-cultured MC38-GFP cells were dissociated using trypsin without EDTA and collected for flow cytometry analysis. For staining, cells were first washed twice with pre-cooled BioLegend’s Cell Staining Buffer and then resuspended in Annexin V Binding Buffer to a concentration between 0.25 and 1.0 × 10^7^ cells/ml. A 100 µl portion of the cell suspension was transferred to a 5 ml tube, to which 5 µl of APC Annexin V was added, followed by 5 µl of 7-AAD Viability Staining Solution. The cells were gently vortexed and incubated in the dark at room temperature (25 °C) for 15 min. Subsequently, 400 µl of Annexin V Binding Buffer was added to each tube. Flow cytometry analysis was performed using appropriate settings with red laser excitation (633 nm). The apoptosis staining kit used was the APC Annexin V Apoptosis Detection Kit with 7-AAD (640930, Biolegend).

Co-cultured MC38-GFP cells were dissociated using trypsin (without EDTA) and collected for flow cytometry analysis. For staining, the cells were first washed twice with pre-cooled BioLegend Cell Staining Buffer and then resuspended in Annexin V Binding Buffer to a concentration of 0.25–1.0 × 10^7^ cells/ml. A 100 µl aliquot of the cell suspension was transferred to a 5 ml tube, to which 5 µl of APC Annexin V was added, followed by 5 µl of 7-AAD Viability Staining Solution. The cells were gently vortexed and incubated in the dark at room temperature (25 °C) for 15 min. Subsequently, 400 µl of Annexin V Binding Buffer was added to each tube. Flow cytometry analysis was performed using appropriate instrument settings with red laser excitation (633 nm). The APC Annexin V Apoptosis Detection Kit with 7-AAD (catalog no. 640930, BioLegend) was used for staining.

### ELISA

The concentration of osteopontin (OPN) in mouse serum samples was quantified using the Mouse/Rat Osteopontin ELISA Kit (KE10046; Proteintech) with the following procedure. The kit and samples were first equilibrated to room temperature (RT) for 20 min, serum samples were diluted 1:100 with PBS prior to analysis. Next, 100 μl of standards or samples was added to each well, and the plate was incubated at 37 °C for 120 min. After draining the liquid from each well, 100 μl of the Biotinylated Detection Antibody working solution was added, and the plate was incubated for 60 min at 37 °C. The wells were then washed three times with wash buffer, followed by the addition of 100 μl of HRP Conjugate working solution and incubation for 40 min at 37 °C. After washing the wells five times with wash buffer, 100 μl of Substrate Reagent was added, and the plate was incubated at 37 °C for approximately 15 min. Finally, 100 μl of Stop Solution was added, and the optical density (OD) was measured at 450 nm. All data were analyzed using ELISACalc.

### Antibody-mediated depletion of intratumoral γδ T cells in MC38 tumor-bearing mice

Female C57BL/6 mice (6–8 weeks old) were housed in a specific pathogen-free (SPF) animal facility. Beginning on day −14, the mice received daily drinking water either containing an antibiotic cocktail (ATBs) or lacking antibiotics. On day 0, MC38 tumor cells were subcutaneously inoculated. Starting on day 4, mice were administered intraperitoneal injections of 300 µl anti-mouse TCR γ/δ (BE0070, clone UC7-13D5, BioXcell) or an isotype control (Armenian hamster IgG, BE0091, BioXcell) for a total of five injections. From day 7 onward, mice received intraperitoneal injections of 200 µg of anti-PD-1 monoclonal antibody (clone RMP1-14, BioXcell) or an isotype control (clone 2A3, BioXcell) every 3 days for a total of four doses. Subcutaneous tumors were collected on day 17.

### Univariate Cox analysis of Spp1 expression across TCGA cancer cohorts

To explore the prognostic relevance of *SPP1* expression, we performed univariate Cox proportional hazards analysis across all available TCGA cancer cohorts. Gene expression matrices (in Transcripts Per Million, TPM) and overall survival (OS) data were downloaded from XenaHub. For each cohort, *SPP1* TPM values were correlated with OS data using univariate Cox analysis. Hazard ratios, along with their associated *p*-values and 95% confidence intervals (CIs), were subsequently visualized in forest plots to provide an aggregate view of *SPP1*’s prognostic significance across multiple cancer types.

### Survival analysis

The prognostic performance of specific tumor-infiltrating cell types and the glycolysis score were evaluated in two immunotherapy cohorts: BLCA and melanoma. The proportions of specific tumor-infiltrating cell types and the glycolysis signature score were calculated as described above. Patients in each cohort were stratified into high- and low-risk groups based on the optimal cutpoint determined by the surv_cutpoint function. The impact of these variables on overall survival was then assessed using the Cox proportional hazards model implemented in the R package survival. Survival curves were generated using the Kaplan–Meier method and visualized with the ggsurvplot function from the survminer package.

## Supplementary information

supplementary materials

## Data Availability

The scRNA-seq matrix data was deposited in https://zenodo.org/records/15014284 (10.5281/zenodo.15014284), and 16S sequencing microbiota data was deposited in https://www.ncbi.nlm.nih.gov/sra/ (ID: PRJNA1231706).
