# Supplementary material for: Single-cell transcriptomic analysis reveals gut microbiota-immunotherapy synergy through modulating tumor microenvironment
Source: Signal Transduct Target Ther. 2025 May 2;10:140. doi: 10.1038/s41392-025-02226-7 (PMC12045981; doi:10.1038/s41392-025-02226-7)
Supplement: Supplementary file 1 — supplementary materials [file 41392_2025_2226_MOESM1_ESM.docx]

Supplementary Materials for

Single-cell transcriptomic analysis reveals gut microbiota-immunotherapy synergy through modulating tumor microenvironment

Minyuan Cao^1,2^*, Yun Deng^2^*, Qing Hao^2^*, Huayun Yan^2^*, Quan-Lin Wang^3^*, Chunyan Dong^2^, Jing Wu^2^, Yajiao He^2^, Li-Bin Huang^4^, Xuyang Xia^1,2,4^, Yongchao Gao^3^, Hai-Ning Chen^5^, Wei-Han Zhang^6^, Yan-Jing Zhang^7^, Xiaozhen Zhuo^8^, Lunzhi Dai^2^, Hongbo Hu^2^, Yong Peng^2^, Feng Zhang^9^, Zhaoqian Liu^3^, Weihua Huang^3^, Huiyuan Zhang^2^, Li Yang^2^, Yang Shu^2,6^, Wei Zhang^3,10#^, Yan Zhang^11#^, Heng Xu^1,2,12,13#^

Correspondence to: [xuheng81916@scu.edu.cn](mailto:xuheng81916@scu.edu.cn) or [zhang.yan@scu.edu.cn](mailto:zhang.yan@scu.edu.cn) or csuzhangwei@csu.edu.cn

**This PDF file includes:**

Supplementary Figures. S1 to S12

**
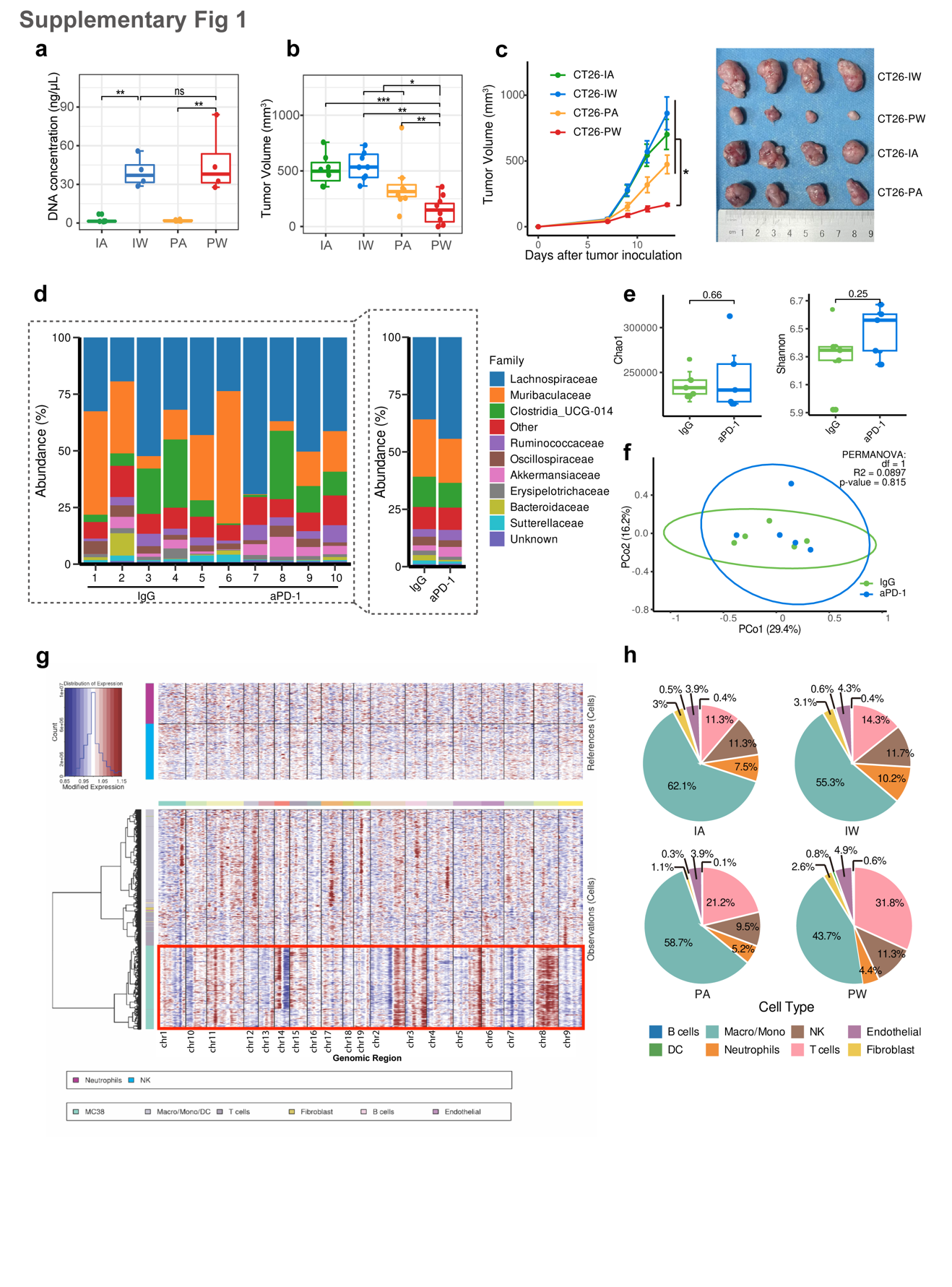
**

**Supplementary Fig 1. Measurement of gut microbiota, tumor volume, and MC38 cells**

1. Box plot showing DNA concentration in fecal samples from the four treatment groups, indicating depletion of gut microbiota after antibiotic (ATB) treatment. Each group consists of 4 mice. Statistical significance was determined using one-way ANOVA (p < 0.05, p < 0.01, *p < 0.001, ns = not significant).
2. Box-and-whisker plot depicts the subcutaneous tumor volumes among four groups. Each group consists of 8 mice (two batches combined).
3. Tumor growth curves for CT26 tumor-bearing mice under different treatment conditions. The right panel shows representative tumor images at the endpoint. Each group consists of 4 mice. Data are represented as mean ± SEM.
4. Bar plot of gut microbiota composition at the family level in different treatment groups. The right inset shows the relative abundance of microbiota in IgG and PD-1 inhibitor-treated groups. Each group consists of 5 mice.
5. Alpha diversity indices (Chao1 and Shannon) comparing gut microbiota diversity between the IgG and PD-1 inhibitor-treated groups. Each group consists of 5 mice.
6. Principal coordinates analysis (PCoA) of beta diversity based on Bray-Curtis distance. Each group consists of 5 mice.
7. Heatmap presenting the inferred copy number variation (CNV) profiles across individual cells from single-cell sequencing data, with major cell types annotated. The red box highlights a subset of MC38 Tumor cells.
8. Pie charts showing the proportions of major cell types in the TME across different treatment groups.

**
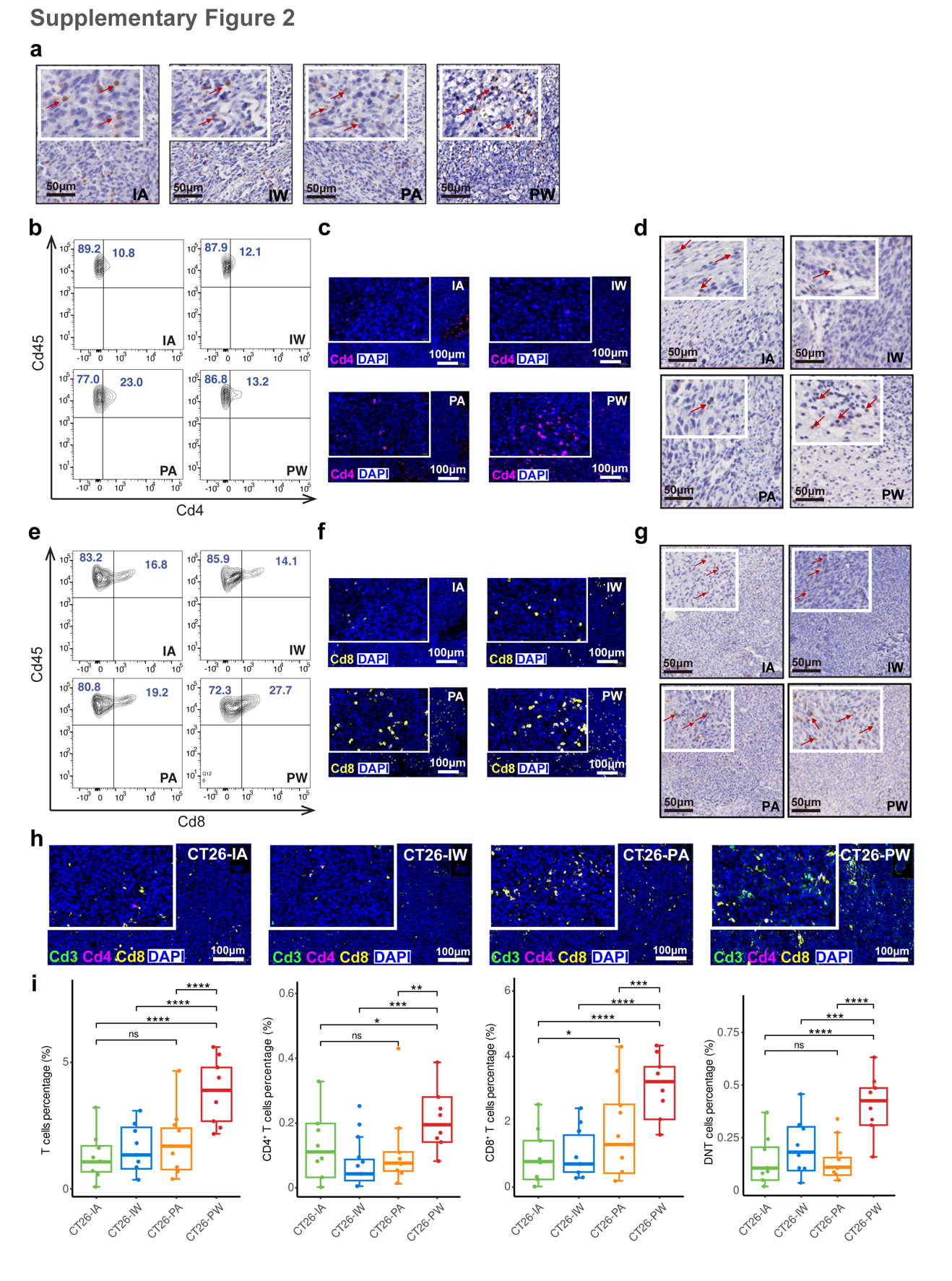
**

**Supplementary Fig 2. Impact of gut microbiota and ICI treatment on proportion of tumor-infiltrating T cells**

1. Representative IHC images of *CD3*⁺ T cells in tumor sections from different treatment groups (IA, IW, PA, PW). Red arrows indicate *CD3*⁺ T cells.
2. Flow cytometry analysis of *CD4*⁺ T cells in tumor tissues from the four treatment groups. The percentage of *CD4*⁺ T cells (CD45⁺CD4⁺) is increased in the PW group compared to other groups.
3. mIF staining for Cd4 (magenta) and DAPI (blue) in tumor sections from different treatment groups.
4. Representative IHC staining for *CD4*⁺ T cells, with red arrows indicating positive cells.
5. Flow cytometry analysis of *CD8*⁺ T cells in tumor tissues from the four treatment groups. The percentage of *CD8*⁺ T cells (CD45⁺CD8⁺) is significantly increased in the PA and PW group.
6. mIF staining for CD8 (yellow) and DAPI (blue) in tumor sections. *CD8*⁺ T cells are more abundant in the PA and PW groups, consistent with flow cytometry data.
7. Representative IHC staining for *CD3*⁺ T cells, with red arrows indicating positive cells.
8. Representative mIF images showing CD3 (green), CD4 (magenta), CD8 (yellow), and DAPI (blue) staining in CT26 tumors from different treatment groups.
9. Box plots quantifying the percentage of total T cells, *CD4*⁺ T cells, *CD8*⁺ T cells, and double-negative T cells (DNT) across different groups. Each group comprises four tumors, with data collected from two sections per tumor. Statistical significance was determined using one-way ANOVA (p < 0.05, p < 0.01, *p < 0.001, **p < 0.0001, ns = not significant).


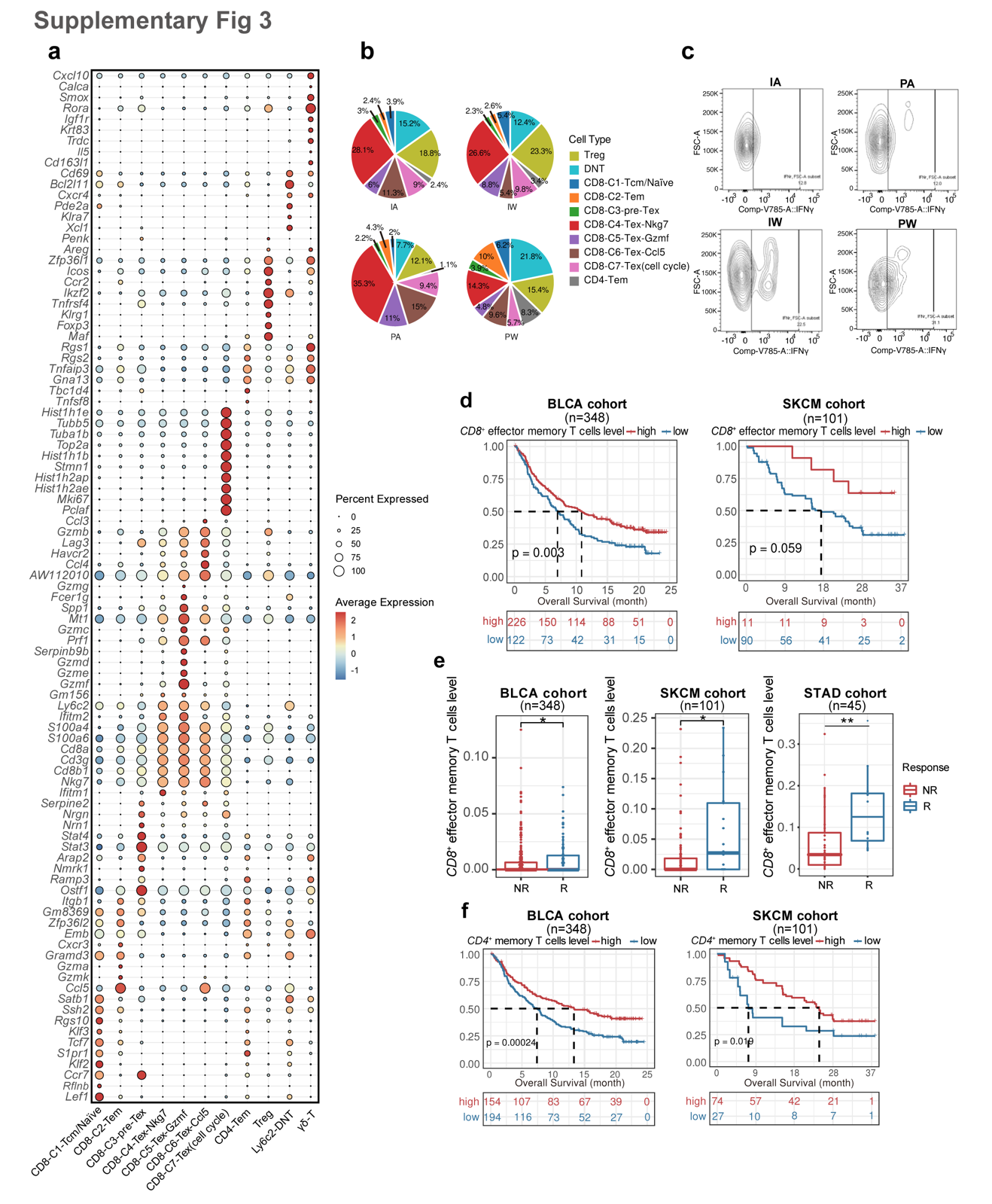


**Supplementary Fig 3. Profile of T cell subtypes and their impact on immunotherapy outcomes**

1. Bubble chart demonstrates the expression profiles of marker genes within *CD8*^+^ T cell subsets.
2. Pie charts illustrating the composition of T cell subtypes in different treatment groups (IA, IW, PA, PW).
3. Flow cytometry chart quantifies the IFN-γ^+^ T cells across four groups.
4. Kaplan-Meier survival curves correlate the CIBERSORT-based proportion of *CD8*^+^ effector memory T cells with ICI treatment outcomes in two immunotherapy cohorts.
5. Box-and-whisker plot compares the CIBERSORT-based proportions of *CD8*^+^ effector memory T cells in tumors from ICI responders with those from non-responders in three immunotherapy cohorts.
6. Kaplan-Meier survival curves correlate the CIBERSORT-based proportions of *Cd4*^+^ memory T cells with ICI treatment outcomes in two immunotherapy cohorts.

**
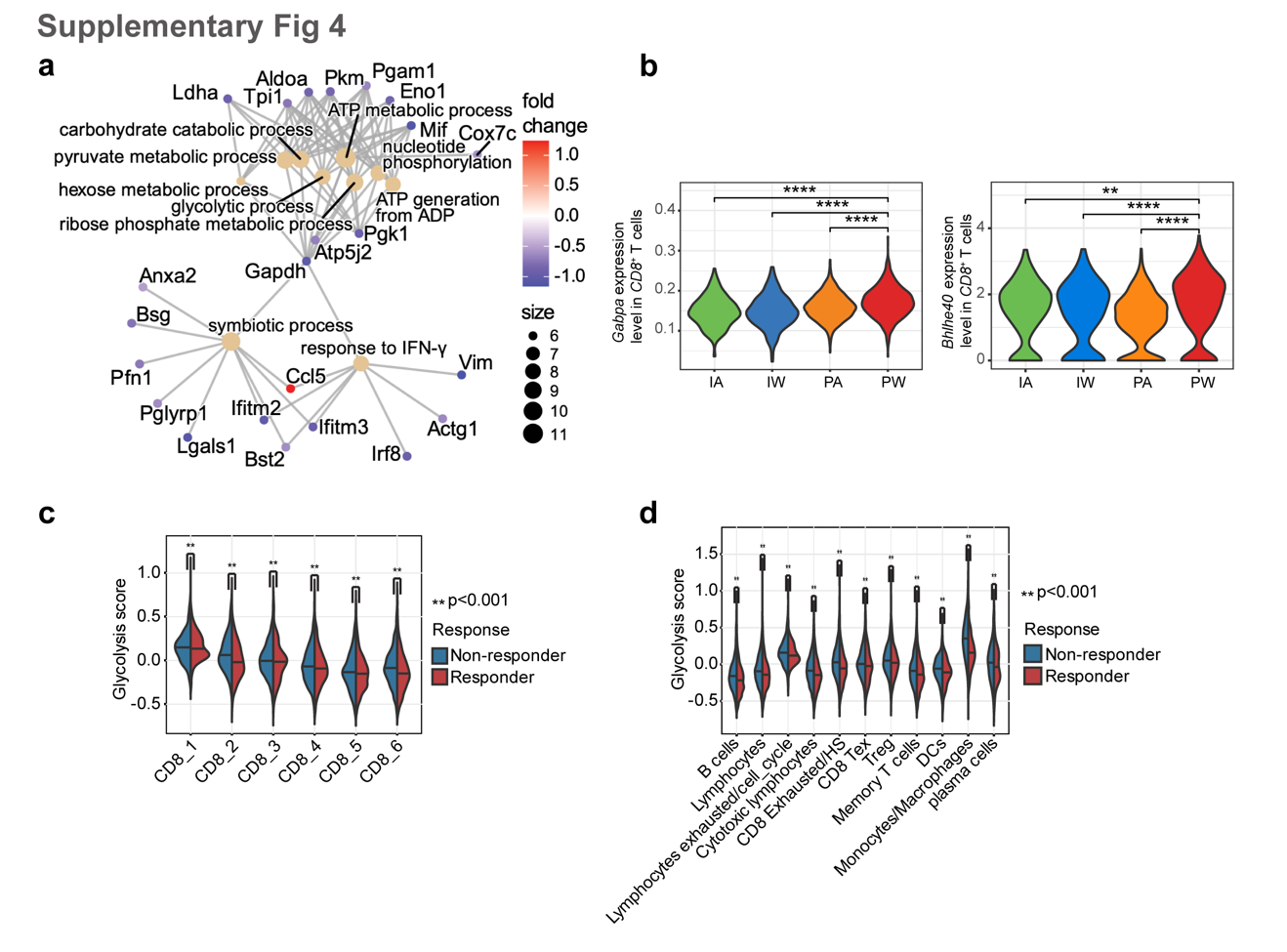
**

**Supplementary Fig 4. Synergistic effects of gut microbiota and ICI treatment on glycolysis**

1. Network diagram depicts the top 10 enriched pathways in *CD8*^+^ T cells influenced by gut microbiota and ICI treatment.
2. Violin plot shows the expression levels of *Gabpa* and *Bhlhe40* in *CD8*^+^ T cells across four groups.
3. Violin plot compares the glycolysis levels in *CD8*^+^ T cells from ICI responders and non-responders based on GSE120575 dataset.
4. Violin plot compares the glycolysis levels in all cell types from ICI responders and non-responders based on GSE120575 dataset.

**
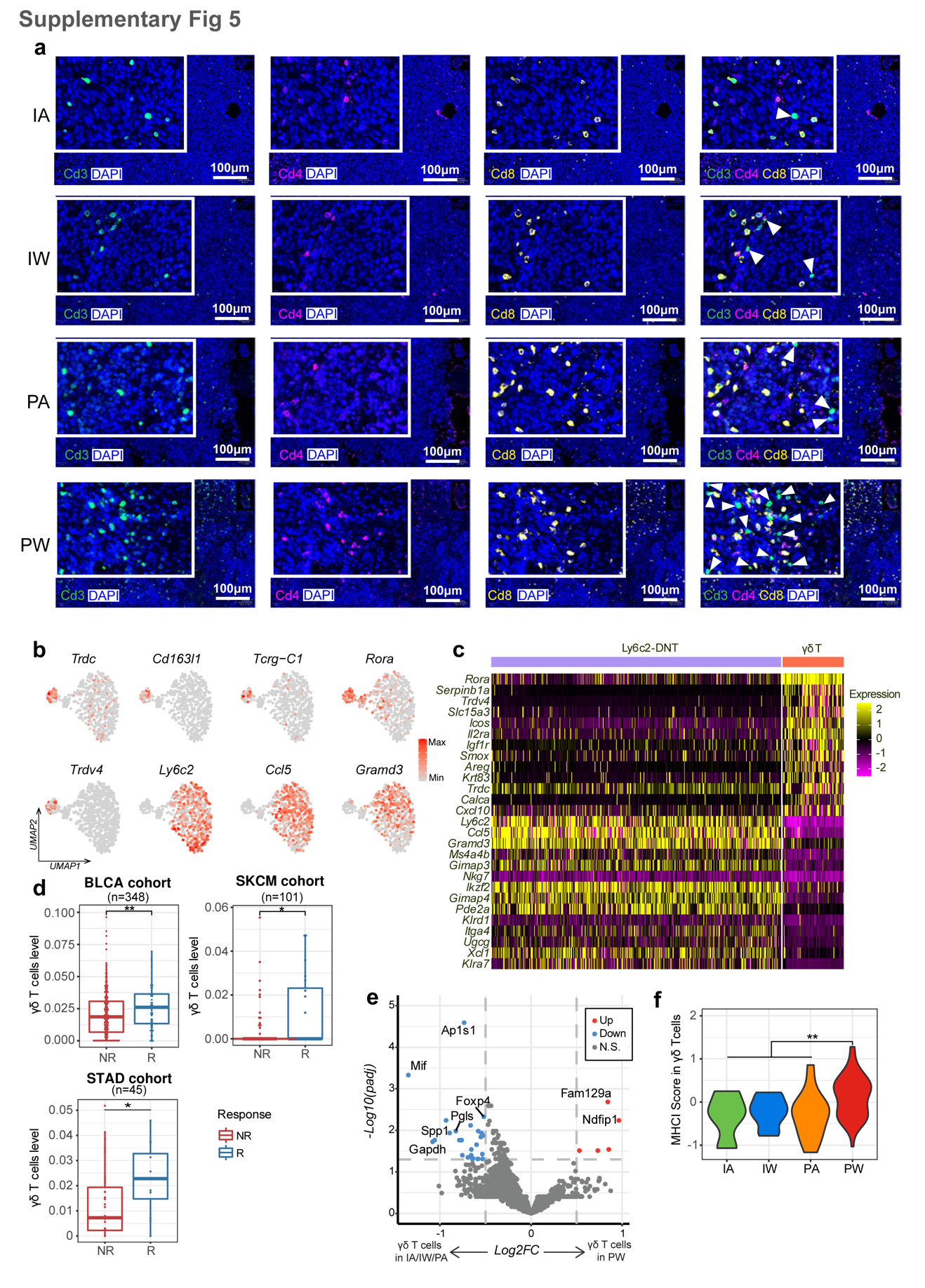
**

**Supplementary Fig 5. Synergistic effect of gut microbiota and ICI treatment on profile of double negative T (DNT) cells**

1. Representative multiplex immunofluorescence (mIF) images illustrate the distribution of *CD4*^+^, *CD8*^+^, and DNT cells in subcutaneous tumors across four groups.
2. UMAP projection illustrates the expression of marker gene in DNT cells.
3. Heatmap representation of the expression levels of relevant marker genes across two DNT cell subtypes.
4. Box plots compare the proportional differences of γδ T cells between ICI treatment responders and non-responders in three immunotherapy cohorts.
5. Volcano plot showcases the differential gene expression in γδ T cells from subcutaneous tumors of the PW group compared to those in the other groups.
6. Violin plot demonstrates the synergistically effect on MHC-I score variability in γδ T cells across four groups.

**
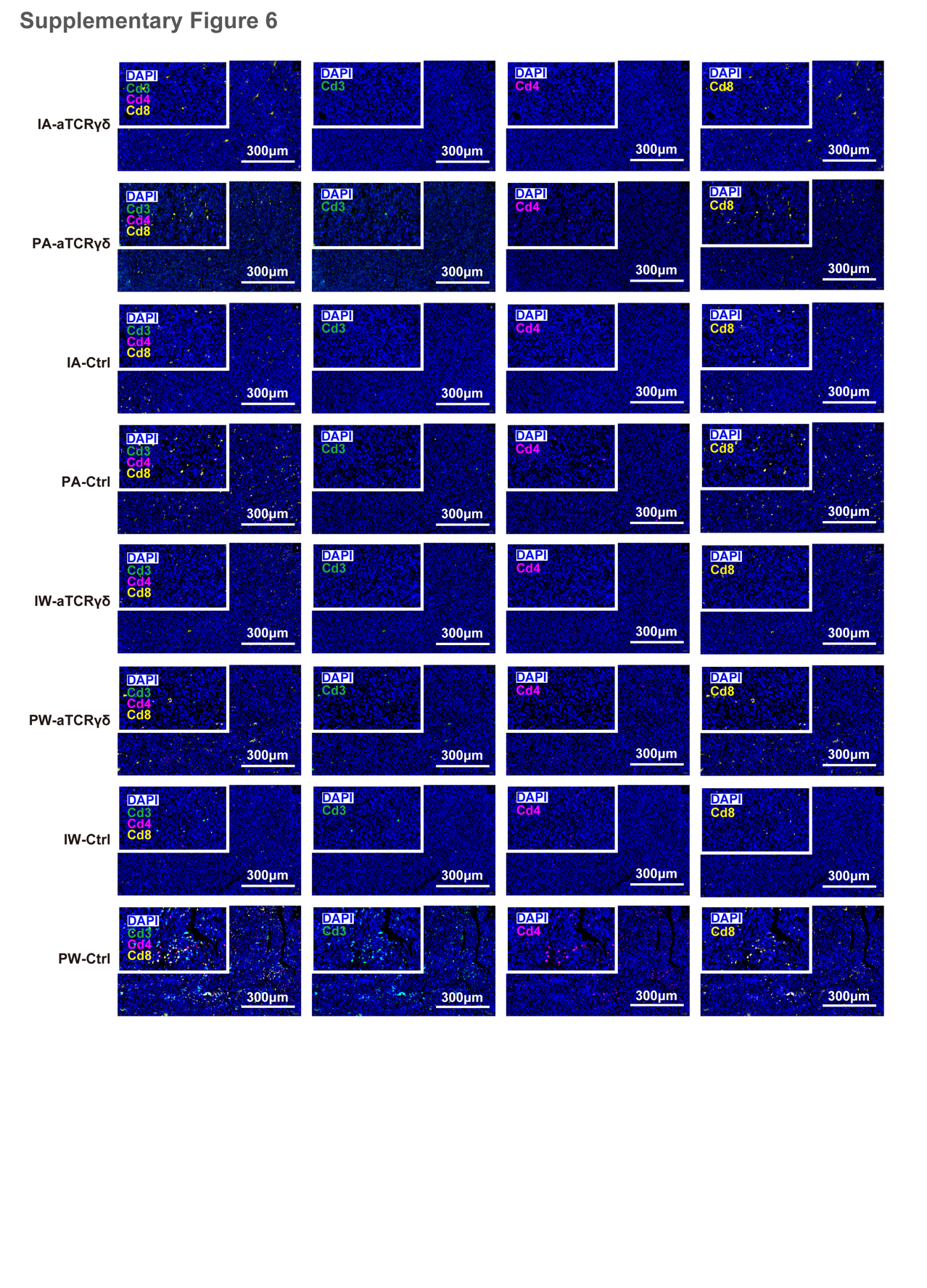
Supplementary Fig 6.** **Depletion of γδ T cells reduces T cell infiltration in the tumor microenvironment.**

Multiplex immunofluorescence (mIF) staining for CD3 (green), CD4 (magenta), CD8 (yellow), and DAPI (blue) in tumor sections from different treatment groups with or without TCRγδ antibody depletion. Groups include IA-aTCRγδ, PA-aTCRγδ, IW-aTCRγδ, PW-aTCRγδ, IA-Ctrl, PA-Ctrl, IW-Ctrl, and PW-Ctrl. TCRγδ antibody treatment led to a significant reduction in γδ T cells, accompanied by a decrease in total infiltrating T cells, particularly in the PW group, which previously exhibited enhanced T cell infiltration. Scale bar = 300 μm.


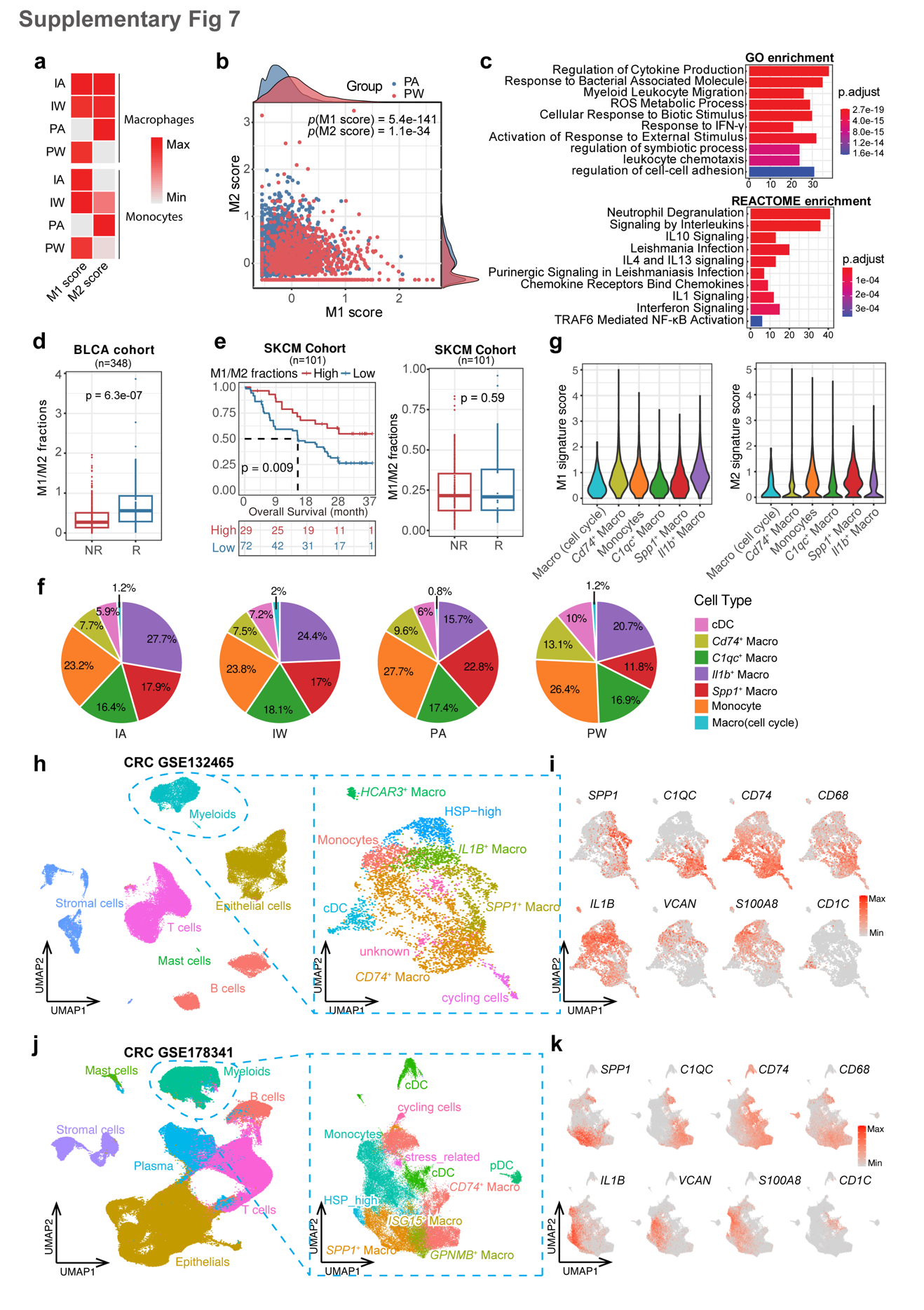


**Supplementary Fig 7. Myeloid profile of mice and colorectal cancer (CRC) patients**

1. Heatmap indicates M1 and M2 signature scores of macrophages/monocytes across four mice groups
2. Marginal density scatter plot illustrates the distribution of M1 and M2 scores for macrophages and monocytes in subcutaneous tumors from PA and PW group mice.
3. Bar graph shows the pathway enrichment of differentially expressed genes (DEGs) in macrophages and monocytes between PW and PA groups in mouse subcutaneous tumors.
4. Box plot compares the proportions of M1/M2 cells ratio between ICI-responders and non-responders in the bladder cancer (BLCA) immunotherapy cohort.
5. Kaplan-Meier survival curve (left) demonstrate the correlation of M1/M2 ratio with immunotherapy treatment outcomes in the melanoma (SKCM) cohort. Box plot (right) compares the proportions of M1/M2 cells ratio between ICI-responders and non-responders in the SKCM immunotherapy cohort.
6. Pie charts illustrating the composition of myeloid cell subtypes in different treatment groups (IA, IW, PA, PW).
7. Violin plots depict M1 and M2 signature scores levels across macrophage subtypes.
8. UMAP visualization reveals the major cell types and myeloid subtypes in CRC GSE132465 validation cohort.
9. Gene expression features plot shows the expression levels of selected marker genes in myeloid cells of CRC GSE132465 validation cohort.
10. UMAP visualization reveals the major cell types and myeloid subtypes in CRC GSE178341 validation cohort.
11. Gene expression features plot shows the expression levels of selected marker genes in myeloid cells of CRC GSE178341 validation cohort.


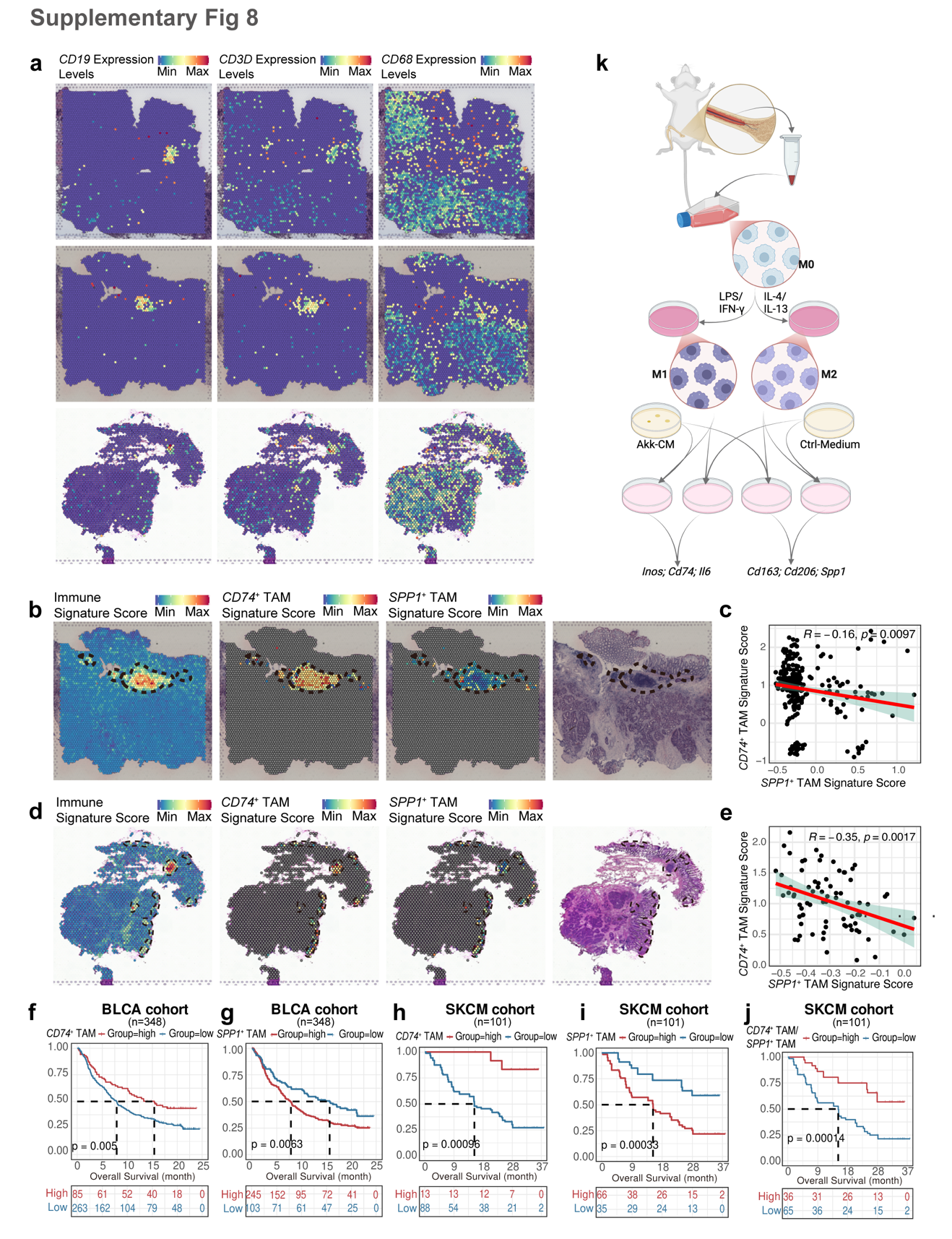


**Supplementary Fig 8. Correlation of *CD74*^+^ tumor associated macrophage (TAMs) and *SPP1*+ TAMs with the special transcriptomic profile**

1. Spatial feature plots display the expression levels of canonical markers for different immune cell types (i.e., B, T, and myeloid cells) from three CRC patients (Qi *et al*., 2022; *Nat Commun* 13:1742).
2. Representative spatial feature plots show the signature score of Immune cells, *CD74*^+^ TAMs and *SPP1*^+^ TAMs in tumor sections from a CRC patient sample (Qi *et al*., 2022; *Nat Commun* 13:1742).
3. Scatter plot reveals the correlation between signature scores of *CD74*^+^ TAMs and *SPP1*^+^ TAMs in immune cell enriched region of the CRC tumor sample in B.
4. Representative spatial feature plots show the signature score of Immune cells, *CD74*^+^ TAMs and *SPP1*^+^ TAMs in tumor sections from a CRC patient sample (Qi *et al*., 2022; *Nat Commun* 13:1742).
5. Scatter plot reveals the correlation between signature scores of *CD74*^+^ TAMs and *SPP1*^+^ TAMs in immune cell enriched region of the CRC tumor sample in D.
6. Kaplan-Meier survival curves demonstrate the proportions of tumor-infiltrating *CD74*^+^ macrophages with ICI treatment outcomes in the bladder cancer (BLCA) immunotherapy cohort.
7. Kaplan-Meier survival curves demonstrate the proportions of tumor-infiltrating *SPP1*^+^ macrophages with ICI treatment outcomes in the bladder cancer (BLCA) immunotherapy cohort.
8. Kaplan-Meier survival curves demonstrate the proportions of tumor-infiltrating *CD74*^+^ macrophages with ICI treatment outcomes in the melanoma (SKCM) immunotherapy cohort.
9. Kaplan-Meier survival curves demonstrate the proportions of tumor-infiltrating *SPP1*^+^ macrophages with ICI treatment outcomes in the melanoma (SKCM) immunotherapy cohort.
10. Kaplan-Meier survival curves demonstrate the proportions of tumor-infiltrating *CD74*^+^ macrophages to *SPP1*^+^ macrophages with ICI treatment outcomes in the melanoma (SKCM) immunotherapy cohort.
11. Illustration summarizing the experimental workflow of in vitro macrophage polarization experiments. Created with BioRender.com.

**
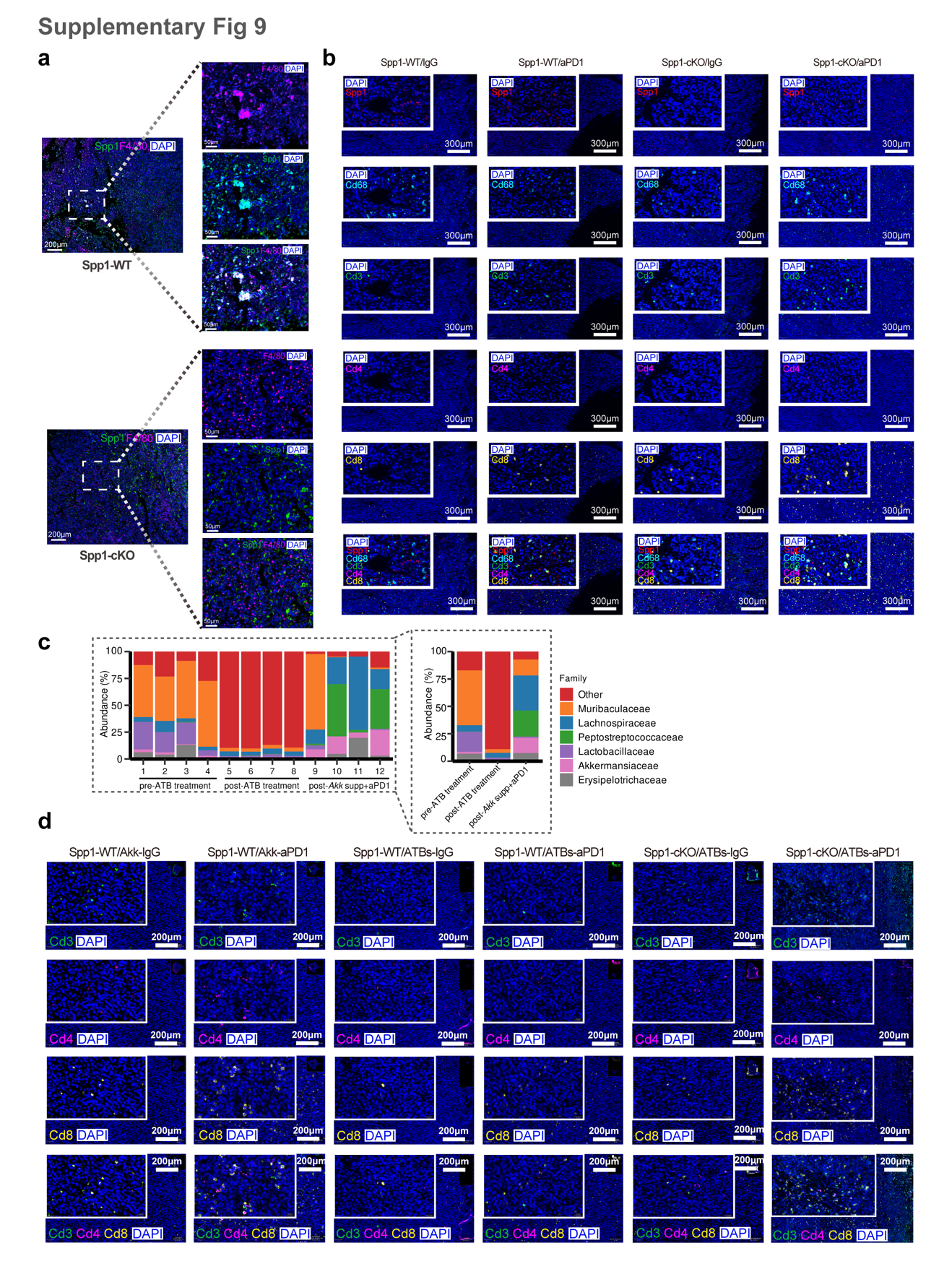
**

**Supplementary Fig 9 TME and gut microbiota profile of Spp1-cKO mice**

1. Representative multiplex immunofluorescence (mIF) images showing SPP1 (green), F4/80 (magenta), and DAPI (blue) in tumor sections from Spp1-WT (wild-type) and Spp1-cKO mice. Insets highlight regions of interest, indicating a significant reduction of SPP1 expression in the Spp1-cKO group.
2. mIF staining of immune markers in tumor sections from Spp1-WT and Spp1-cKO mice under different treatment conditions (IgG control or anti-PD1).
3. Gut microbiota composition analysis before and after antibiotic (ATB) treatment and *Akkermansia muciniphila* (*Akk*) supplementation in Spp1-WT mice. The left panel presents microbiota composition across individual samples, while the right panel shows aggregated data grouped by different time points.
4. mIF staining of CD3 (green), CD4 (magenta), CD8 (yellow), and DAPI (blue) in tumor sections from Spp1-WT and Spp1-cKO mice under different treatment conditions (ATBs + IgG or ATBs + anti-PD1). The Spp1-cKO group exhibits an increase in T cell infiltration, even after gut microbiota depletion.

**
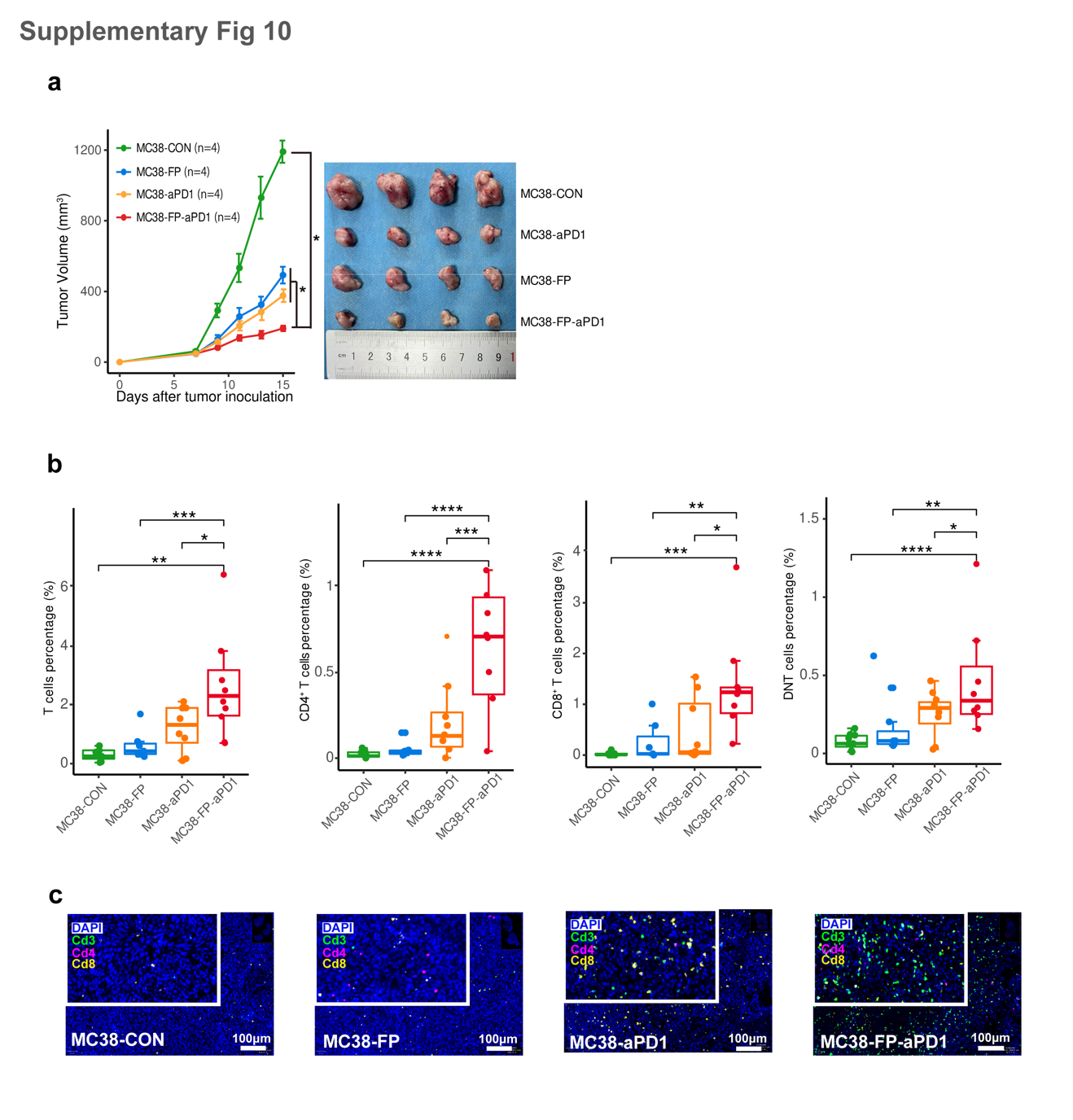
**

**Supplementary Fig 10. Faecalibacterium prausnitzii (FP) supplementation enhances ICI efficacy and promotes T cell infiltration in MC38 tumors.**

1. Tumor growth curves and representative tumor images from MC38 tumor-bearing mice under different treatment conditions. Mice were treated with control (MC38-CON), FP supplementation (MC38-FP), anti-PD1 (MC38-aPD1), or FP combined with anti-PD1 (MC38-FP-aPD1). Tumor volume was significantly reduced in the MC38-FP-aPD1 group, highlighting the synergistic effect of FP supplementation and anti-PD1 therapy.
2. Box plots quantifying the percentage of different tumor-infiltrating T cell populations across treatment groups. Each group comprises four tumors, with data collected from two sections per tumor. Statistical significance was determined using one-way ANOVA (p < 0.05, p < 0.01, *p < 0.001, **p < 0.0001).
3. Representative multiplex immunofluorescence (mIF) images of tumor sections stained for CD3 (green), CD4 (magenta), CD8 (yellow), and DAPI (blue).

**
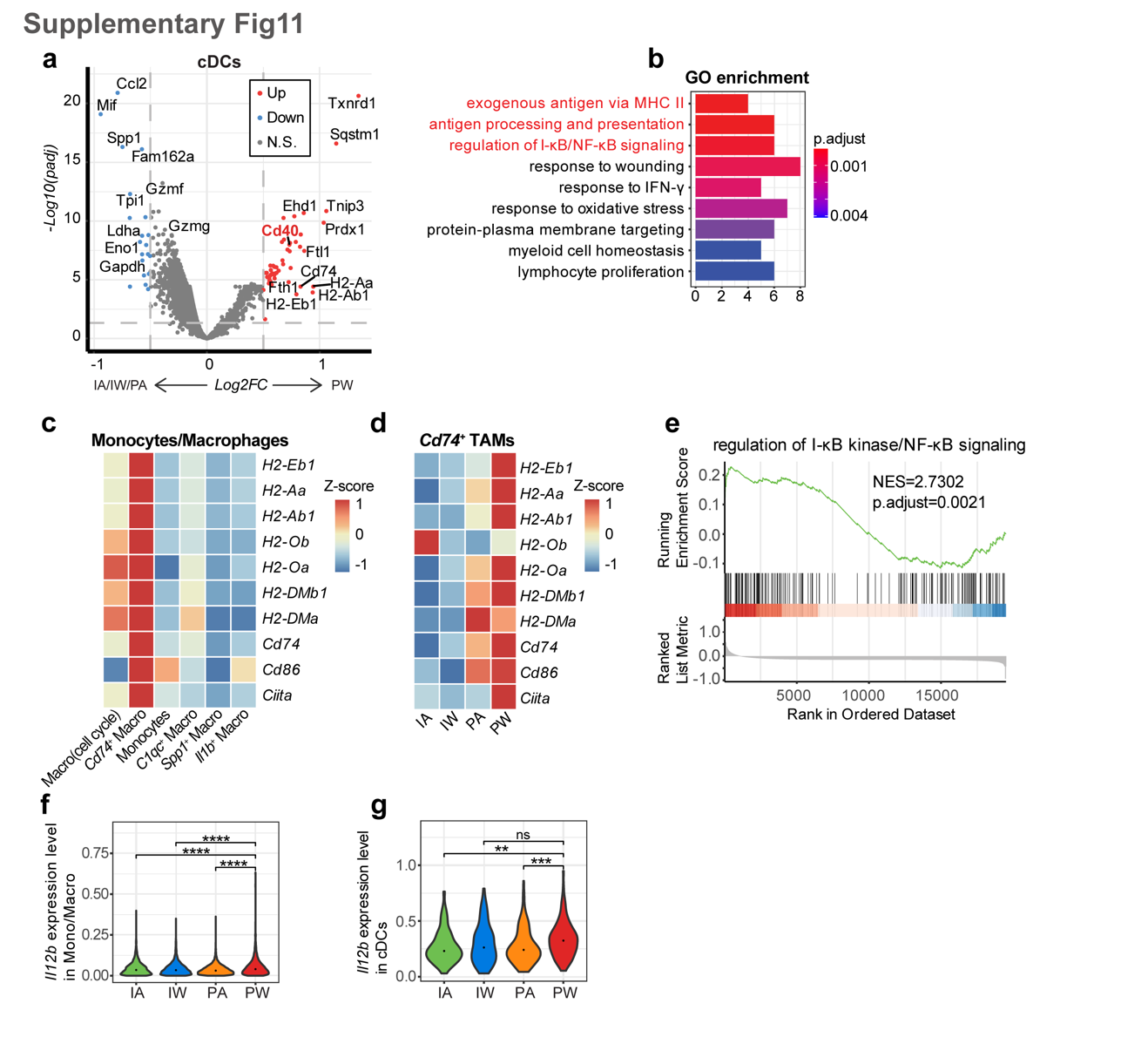
**

**Supplementary Fig 11 Synergistic regulation of antigen presentation and NF-κB signaling in myeloid cells by gut microbiota and PD-1 inhibitor**

1. Volcano plot delineates the differentially expressed genes (DEGs) in conventional dendritic cells (cDCs) from subcutaneous tumors of PW group compared with those from the others.
2. Bar graph shows the pathway enrichment analysis of DEGs as shown in A.
3. Heatmap presents the expression levels of MHC-II-related genes across different myeloid cell subtypes.
4. Heatmap presents the expression levels of MHC-II-related genes in *Cd74*^+^ macrophages across four groups.
5. Gene Set Enrichment Analysis (GSEA) analysis chart exhibits the enrichment of the NF-κB pathway with the DEGs between antigen-presenting cells from the PW group and those from the other three groups.
6. Violin plot illustrates the expression levels of the *Il12b* in monocytes and macrophages across four groups.
7. Violin plot illustrates the expression levels of the *Il12b* in cDCs across four groups.

##
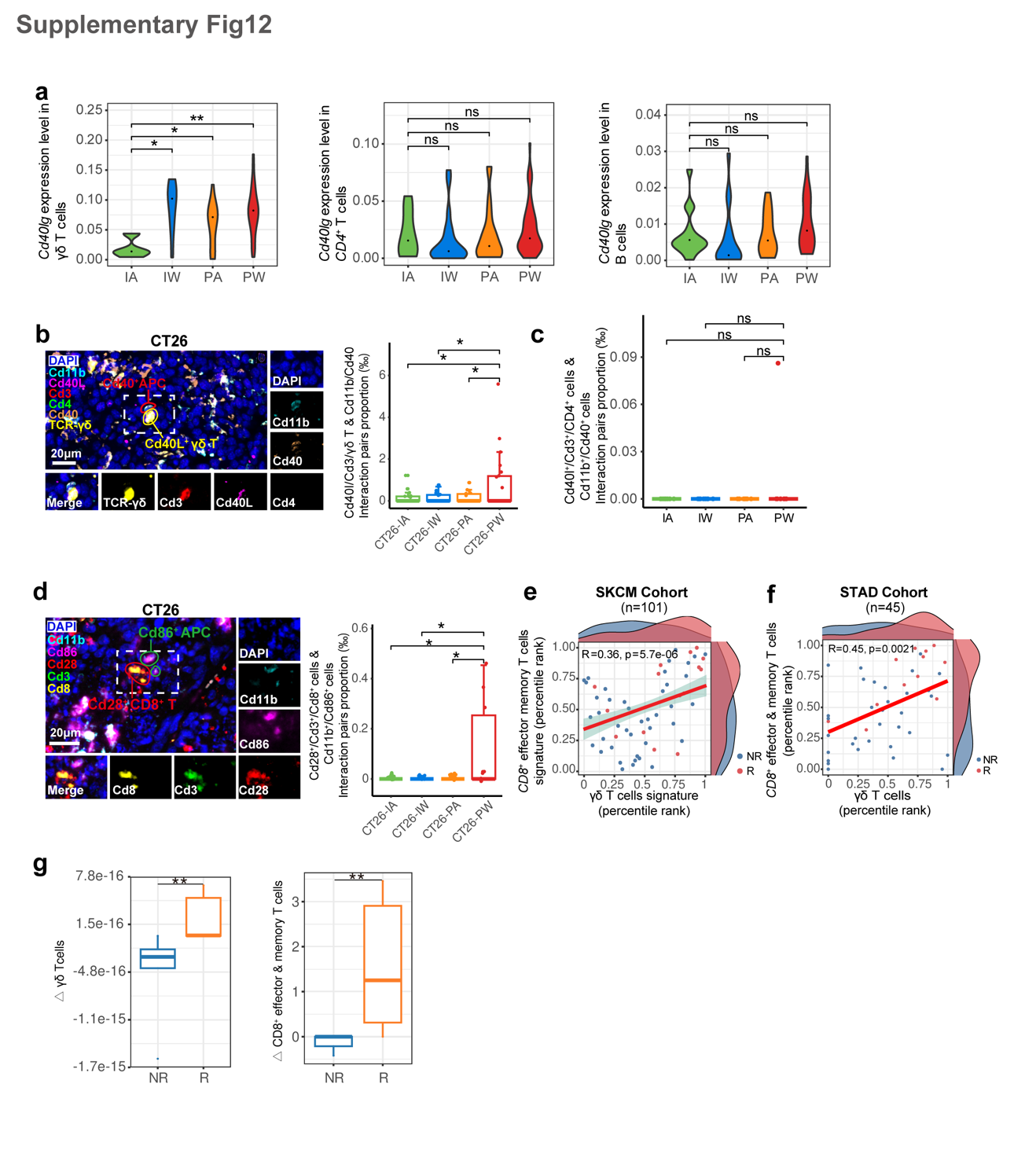
**Supplementary Fig 12. Cd40l-Cd40 mediated γδ T cell-APC-*CD8*^+^ T cell axis enhance *CD8*⁺ effector/memory T cell activation in response to gut microbiota and ICI therapy.**

1. Violin plots showing Cd40lg expression levels in different immune cell subsets (γδ T cells, CD4⁺ T cells, and B cells) across treatment groups (IA, IW, PA, PW). Statistical significance was determined using one-way ANOVA (p < 0.05, p < 0.01, ns = not significant).
2. mIF images and quantification of *Cd40*^+^ APC and *Cd40L*^+^ γδ T cell interactions in CT26 tumors. The left panel shows representative mIF staining for CD40 (brown), CD40L (magenta), TCR-γδ (yellow), CD11b (cyan), CD3 (red), CD4 (green), and DAPI (blue) in tumor sections. Each group comprises four tumors, with data collected from two sections per tumor. The right panel quantifies the proportion of *Cd40*^+^ APC and *Cd40L*^+^ γδ T cell interactions, showing a significant increase in the PW group compared to the other groups.
3. Quantification of *Cd40*^+^ APC and *Cd40L*^+^/*CD4*⁺ T cell interactions across treatment groups. Each group comprises four tumors, with data collected from two sections per tumor.
4. mIF images and quantification of *Cd86*^+^ APC and *Cd28*^+^/*CD8*⁺ T cell interactions in CT26 tumors. The left panel shows representative mIF staining for CD86 (magenta), CD28 (red), CD11b (cyan), CD3 (green), CD8 (yellow), and DAPI (blue). The right panel quantifies the proportion of *Cd86*^+^ APC and *Cd28*^+^/*CD8*⁺ T cell interactions, revealing a significant increase in the PW group. Each group comprises four tumors, with data collected from two sections per tumor.
5. Scatter plots showing the correlation between γδ T cell signatures and *CD8*⁺ effector/memory T cell signatures in independent SKCM immunotherapy cohorts.
6. Scatter plots showing the correlation between γδ T cell signatures and *CD8*⁺ effector/memory T cell signatures in independent STAD immunotherapy cohorts.
7. Box plots showing changes in γδ T cells and CD8⁺ effector/memory T cells post-FMT treatment in responders (R) and non-responders (NR). The dataset includes a total of 9 pairs of samples (3 R and 6 NR).
